# Correction: Prenatal and postnatal determinants of stunting at age 0–11 months: A cross-sectional study in Indonesia

**DOI:** 10.1371/journal.pone.0315015

**Published:** 2024-12-02

**Authors:** The PLOS ONE Editors

## Notice of Republication

This article [1] was republished on November 1, 2024, to address an issue identified post-publication. The Data Availability statement is updated to: The data belong to the Research Unit SEAMEO RECFON and access is restricted for ethical and legal reasons. Data access will be granted once users have consented to the data sharing agreement and provided written plans and justification for their proposed use of the data. Data access may be obtained by submitting a request to the Research Unit SEAMEO RECFON (research@seameo-recfon.org) and will be reviewed by SEAMEO RECFON. Data sharing is subject to the Regulation of the Ministry of Health of the Republic of Indonesia no. 85/2020, on Data Sharing.

Please download this article again to view the correct version.
