# Supplementary material for: Prenatal and postnatal determinants of stunting at age 0–11 months: A cross-sectional study in Indonesia
Source: PLoS One. 2021 Jul 14;16(7):e0254662. doi: 10.1371/journal.pone.0254662 (PMC8279365; doi:10.1371/journal.pone.0254662)

Kecamatan | Desa | No. Responden

SURVEI HUBUNGAN STATUS GIZI IBU SELAMA KEHAMILAN DENGAN DAMPAK KELAHIRAN DAN STATUS GIZI ANAK USIA 0-11 BULANDI KABUPATEN SAMBAS, KALIMANTAN BARAT- FOLLOW UP STUDY

GIZI KOMUNITAS, FAKULTAS KEDOKTERAN
SEAMEO RECFON, UNIVERSITAS INDONESIA
Jl. Salemba Raya No 6, 10430, JAKARTA
Tlp. +62 (21) 390 9205 , Fax. +62 (22) 391 3933

FORM WAWANCARA

Tim Survei [SRVY_TM]

01 : Tim A   02 : Tim B   03 : Tim C

Kode Pewawancara [enum]

01 Annisa Dwi Utami
02 Arindah Nur Sartika
03 Fitriana Nugrahaeni
04 Grace Sheila Jonatan
05 Khalida Fauzia
06 Laila Mardiana
07 Meirina Khoirunisa
08 Nurul Hasanah
09 Rahmawati
10 Wanda Lasepa
11 Afifah
12 Rian
13 Defy
14 Misnadi
15 Hari

Kode Supervisor [spv]

01 :Evi Ermayani   02 : Aziz Jati Nur Ananda   03 :Indriya Laras Pramesthi   04: Edy Waliyo

Keterangan	Isian	Kode	Keterangan	Isian
Tanggal Interview (tgl-bln-thn)		[survyDate]	TandaTangan Pewawancara	
Waktu Survei Mulai		[timeStart]	Nama Ketua Tim	
Waktu Survey Selesai		[timeFinish]		
Tanggal Pemeriksaan[1] (tgl-bln-thn)		[checkDate]	Tanda Tangan Supervisor	
Tanggal Pengumpulan[2] (tgl-bln-thn)		[collctDate]	Tanda Tangan Ketua Tim	
Tanggal Penyerahan[3] (tgl-bln-thn)		[delivDate]	Tanda Tangan Koordinator Data Management	
Tanggal Data Entry[4] (tgl-bln-thn)		[EntryDate]	Tanda Tangan Data Entry	

[1]Supervisor [2]Ketua Tim [3]Koordinator Data Management [4]Petugas Data Entry

Kecamatan	Desa	No. Responden
[ ][ ]	[ ][ ]	[ ][ ][ ]

B. Identitas Responden		Kode	
Nama Pengasuh		[nameCG] <string>	
Hubungan Pengasuh dengan anak	1. Ibu 4. Kakek<br>2. Ayah 5. Kakak<br>3. Nenek 77. Lainnya,_________	[ ]	[relationCG]
Alamat	RT/RW/Dusun: ___/___/_____________		[addrs]
Nomor Telepon			[noHp]
**Kecamatan**			
01. Galing<br>02. Jawai<br>03. Jawai Selatan<br>04. Paloh	05. Pemangkat<br>06. Sajad<br>07. Sajingan Besar<br>08. Salatiga<br>09. Sambas<br>10. Sebawi<br>11. Sejangkung<br>12. Selakau<br>13. Selakau Timur<br>14. Semparuk<br>15. Subah<br>16. Tangaran<br>17. Tebas	[ ]	[subDstrct]
**Desa**			
01. Galing<br>02. Sarang Burung Kuala<br>03. Bakau<br>04. Pelimpaan<br>05. Sarilaba B<br>06. Semperiuk B<br>07. Kalimantan<br>08. Nibung<br>09. Sebubus	10. Perapakan<br>11. Harapan<br>12. Jirak<br>13. Sanatab<br>14. Salatiga<br>15. Tanjung Bugis<br>16. Lumbang<br>17. Semanjang<br>18. Sempalai Sebedang<br>19. Sepuk Tanjung<br>20. Sulung<br>21, Perigi Limus<br>22. Sendoyan<br>23. Kuala<br>24. Gelik<br>25. Sepinggan<br>26. Tebuah Elok<br>27. Tangaran<br>28. Semata<br>29. Segedong<br>30. Sejiram	[ ]	[VILL]

C. Data Pribadi Anak			Kode	
1	Status anak **(PERTANYAAN SENSITIF)**	1. Meninggal<br>2. Hidup → **lanjut ke C2**	[ ]	[chidStat]
**C1. Data untuk anak yang meninggal**				
2	Apa penyebab anak ibu meninggal?	1. Kelainan,sebutkan _________<br>2. Penyakit, sebutkan__________<br>3. Kecelakaan, sebutkan________<br>66. tidak relevan jika anak masih hidup<br>77. lainnya,__________________	[ ]	[mortRsn]
3	Jika sudah meninggal, apakah meninggal dalam kandungan?	1. Ya → **lanjut ke C1.1**<br>0. Tidak → **lanjut ke C1.2**<br>66. Tidak relevan jika anak masih hidup	[ ]	[mortFet]
**C1.1 Data untuk anak yang meninggal dalam kandungan**				
4	Berapa usia kandungan Ibu saat bayi meninggal?	1. Trimester I<br>2. Trimester II<br>3. Trimester III<br>66. Tidak relevan jika anak masih hidup	[ ]	[mortAge]
5	Dimana penanganan terhadap ibu dilakukan?	1. Polindes<br>2. Poskesdes<br>3. Puskesmas kecamatan<br>4. RSUD<br>5. Klinik bidan swasta<br>6. Klinik dokter swasta<br>7. Rumah sakit swasta<br>8. Dukun<br>10. Rumah<br>66. Tidak relevan jika anak hidup<br>77. lainnya,________	[ ]	[bornPlac]
6	Siapa yang menangani Ibu?<br><br>**(WAWANCARA DIHENTIKAN SETELAH PERTANYAAN INI)**	1. Bidan desa<br>2. Bidan puskesmas kecamatan<br>3. Bidan RSUD<br>4. Dokter puskesmas kecamatan<br>5. Dokter RSUD<br>6. Bidan praktek swasta<br>7. Dokter praktek swasta<br>8. Dukun<br>66. Tidak relevan jika anak hidup<br>77. lainnya, ___________	[ ]	[bornHlpr]
**C1.2 Data untuk anak yang meninggal setelah lahir**				
7	Nama Anak			[nameMCH] <string>
8	Jenis Kelamin Anak	1. Laki-laki 2. Perempuan<br>66. Tidak relevan jika anak hidup	[ ]	[childMSex]

Kecamatan		Desa		No. Responden		

9	Tanggal Lahir Anak (dd/mm/yy)			[childMDOB]
10	Pada usia berapa anak Ibu meninggal?	1. Usia ≤ 28 hari<br>2. Usia > 28 hari<br>66. Tidak relevan jika anak hidup	[ ]	[mortAge]
11	Berapakah berat badan anak Ibu ketika dilahirkan?	1. < 2500 g<br>2. ≥ 2500 g<br>66. tidak relevan jika anak hidup<br>88. Tidak tahu	[ ]	[childMBW]
12	Sumber informasi berat badan anak	1. Interview dengan responden<br>2. Buku KIA/KMS<br>3. Tenaga kesehatan (bidan, kader,dll)<br>66. tidak relevan jika anak hidup	[ ]	[MBWinfo]
13	Setelah anak lahir, apakah dilakukan pemeriksaan kesehatan?	0. Tidak<br>1. Ya<br>66. tidak relevan jika anak hidup<br>88. Tidak tahu	[ ]	[MUtilizeHS]
14	Apakah Ibu mendapat pelayanan (neonatal) kesehatan (dikunjungi/mengunjungi) pada	a. 6 – 48 jam setelah lahir<br>b. 3 – 7 hari setelah lahir<br>c. 8 – 28 hari setelah lahir<br><br>1. Ya<br>0. Tidak<br>66. Tidak Relevan, jika meninggal <6 jam, dan masih hidup<br>88. Tidak Tahu	[ ]<br>[ ]<br>[ ]	[NCchildM1]<br>[NCchildM2]<br>[NCchildM3]
15	Siapa yang memeriksakan kesehatan anak saat itu?<br>**(MENGACU JAWABAN NO 14)**	1.Bidan desa<br>2.Bidan puskesmas kecamatan<br>3.Bidan RSUD<br>4.Dokter puskesmas kecamatan<br>5. Dokter RSUD<br>6. Bidan praktek swasta<br>7. Dokter praktek swasta<br>8. Dukun<br>66. tidak relevan<br>77. lainnya, ___________	[ ]<br>[ ]<br>[ ]	[NCofficerM1]<br>[NCofficerM2]<br>[NCofficerM3]
16	Dimana pemeriksaan kesehatan anak dilakukan saat itu?<br>**(MENGACU JAWABAN NO 14)**<br><br>**(WAWANCARA DIHENTIKAN SETELAH PERTANYAAN INI)**	1. Polindes<br>2. Poskesdes<br>3. Puskesmas kecamatan<br>4. RSUD<br>5. Klinik bidan swasta<br>6. Klinik dokter swasta<br>7. Rumah sakit swasta<br>8. Dukun<br>9. Rumah<br>66. Tidak relevan jika anak hidup<br>77. lainnya, ___________	[ ]<br>[ ]<br>[ ]	[NCplaceM1]<br>[NCplaceM2]<br>[NCplaceM3]
**C2. Data Pribadi Anak yang Hidup**				
17	Nama Anak			[nameCH] <string>
18	Jenis Kelamin Anak	1. Laki-laki 2. Perempuan<br>66. Tidak relevan jika anak sudah meninggal	[ ]	[childSex]
19	Tanggal Lahir Anak (dd/mm/yy)			[childDOB]
20a	Berat Lahir	__ __ __ __ gram		[childBW]
20b	Sumber informasi berat badan anak	1. Interview dengan responden<br>2. Buku KIA/KMS<br>3. Tenaga kesehatan (bidan, kader,dll)<br>66. tidak relevan jika anak sudah meninggal	[ ]	[BWinfo]
21	Usia Kandungan saat melahirkan	1. Prematur (<37 minggu)<br>2. Tidak prematur (≥ 37 minggu)<br>66. tidak relevan jika anak sudah meninggal	[ ]	[premature]
22.	Kondisi Ibu kandung	1. Meninggal<br>2. Bekerja/pindah<br>66. tidak relevan jika ibu adalah pengasuh→**lanjut ke 24**<br>77. lainnya, ___________	[ ]	[MotherStatus]
23.	Nama Ibu Kandung			[nameMthr] <string>
24.	Nama Kepala Keluarga			[nameHeadHH] <string>
25.	Hubungan Kepala Keluarga dengan anak	1. Ibu<br>2. Ayah<br>3. Nenek<br>4. Kakek<br>5. Kakak<br>77. Lainnya,_________	[ ]	[relationHeadHH]

Kecamatan		Desa		No. Responden		

	C3. Data Ibu yang Meninggal			
26.	Waktu meninggal ibu kandung	1.saat hamil<br>2. saat melahirkan<br>3. setelah melahirkan<br>66. tidak relevan jika ibu masih hidup<br>→**lanjut ke D**<br>88. tidak tahu<br>99. tidak jawab	[ ]	[MthrMortTime]
27.	Penyebab meninggal ibu kandung	1. Pendarahan<br>2. Hipertensi<br>3. Lainnya, ___________<br>66. Tidak relevan jika ibu masih hidup<br>88. tidak tahu<br>99. tidak jawab	[ ]	[MthrMortRsn]

D. Data Sosial-Demografi			Kode	
1	Tipe keluarga?	1. Keluarga inti 2. Keluarga besar	[ ]	[typFam]
2	Isi data tabel dengan data yang sesuai dengan keadaan rumah tangga			

No	Nama Anggota Keluarga [nameHHmmber]	Hubungan Dengan Anak [relationU1]	Jenis Kelamin [sex]	Usia/Tanggal Lahir [ageHH]	Pekerjaan Utama [occupHH]
1		[ ]	[ ]		[ ]
2		[ ]	[ ]		[ ]
3		[ ]	[ ]		[ ]
4		[ ]	[ ]		[ ]
5		[ ]	[ ]		[ ]
6		[ ]	[ ]		[ ]
7		[ ]	[ ]		[ ]
8		[ ]	[ ]		[ ]
9		[ ]	[ ]		[ ]
10		[ ]	[ ]		[ ]

Note:

Hubungan dengan anak :
1. Anak usia 0-11 bulan
2. Ibu
3. Ayah
4. Nenek
5. Kakek
6. Kakak

77. Lainnya(Sebutkan)

Jenis Kelamin
1. Laki-laki
2. Perempuan

Pekerjaan saat ini :
1. Petani
2. Buruh Tani
3. Buruh (penambang/ pekerja bangunan)
4. Nelayan
5. Peternak
6. PNS
7. Pegawai Honorer
8. Pegawai Swasta
9. Pengusaha
10. Penjual Toko
11. Penjual keliling
12. TKI/TKW
13. Transportation service (ojek, masinis, supir, pengemudi)
14. Ibu Rumah Tangga
15. Pelajar
16. Pengrajin
17. Pensiunan
18. Tidak Bekerja

66. tidak relevan jika belum bekerja
77. Lainnya (sebutkan)
88. Tidak tahu

3	Jumlah anggota keluarga		[ ]	[numHHmem]
4	Jumlah anak < 2 tahun		[ ]	[numChildU2]
5	Jumlah anak 2-5 tahun		[ ]	[numChildU5]
6	Apa status pernikahan ibu kandung?	1. Menikah 3. Cerai atau janda<br>2. Tidak menikah	[ ]	[Marital]
7	Riwayat berat badan lahir rendah **anak sebelumnya**?	0. Tidak 1. Ya (≤2500 gr)<br>88. tidak tahu<br>66. Tidak relevan, jika anak pertama	[ ]	[historyLBW]

Kecamatan	Desa	No. Responden
[ ][ ]	[ ][ ]	[ ][ ][ ]

F.Pemanfaatan Pelayanan Kesehatan				
**F1. Setelah Kelahiran anak**				
1	Dimana anak dilahirkan?	1. Polindes<br>2. Poskesdes<br>3. Puskesmas kecamatan<br>4. RSUD<br>5. Klinik bidan swasta<br>6. klinik dokter swasta<br>7. Rumah sakit swasta<br>8. Dukun<br>10. Rumah<br>66. Tidak relevan jika anak hidup<br>77. lainnya.__________	[ ]	[bornPlace]
2	Siapa yang menangani proses kelahiran ibu? (siapa **NAMA** tenaga kesehatannya)	1. Bidan desa<br>2. Bidan puskesmas kecamatan<br>3. Bidan RSUD<br>4. Dokter puskesmas kecamatan<br>5. Dokter RSUD<br>6. bidan praktek swasta<br>7. Dokter praktek swasta<br>8. Dukun<br>66. Tidak relevan jika anak hidup<br>77. lainnya, ______	[ ]	[bornHlpr]
3.	Setelah anak ini lahir, apakah dilakukan pemeriksaan kesehatan?	0. Tidak<br>1. Ya<br>66. Tidak relevan jika anak meninggal dalam kandungan atau < 6 jam<br>88. Tidak tahu	[ ]	[UtilizeHS]
4.	Setelah kelahiran, apakah ibu (kandung) pernah melakukan kunjungan neonatal ke fasilitas kesehatan? (atau dikunjungi untuk pemeriksaan)			[NCchild1]<br>[NCchild2]<br>[NCchild3]<br>[NCofficer1]<br>[NCofficer2]<br>[NCofficer3]<br>[NCplace1]<br>[NCplace2]<br>[NCplace3]

No.	Periode Kunjungan Neonatal	Dilaksanakan:	Petugas Pemeriksa:	Tempat Pemeriksaan:
1.	KN1: 6-48 jam (sampai 2 hari setelah melahirkan)	[ ]	[ ]	[ ]
2.	KN2: 3-7 hari	[ ]	[ ]	[ ]
3.	KN3: 8-28 hari	[ ]	[ ]	[ ]

Dilaksanakan:
1. Ya
0. Tidak
55. tidak ingat
66. tidak relevan jika jawaban no. 3 adalah “tidak”, atau bayi meninggal <6 jam
88. tidak tahu
99. tidak jawab

Petugas Pemeriksa:
1.Bidan desa
2.Bidan puskesmas kecamatan
3.Bidan RSUD
4.Dokter puskesmas kecamatan
5. Dokter RSUD
6. Bidan praktek swasta
7. Dokter praktek swasta
8. Dukun
66. Tidak relevan
77. lainnya, ___

Tempat Pemeriksaan:
1. Polindes
2. Poskesdes
3. Puskesmas kecamatan
4. RSUD
5. Klinik bidan swasta
6. Klinik dokter swasta
7. Rumah sakit swasta
8. Dukun
9. Rumah
66. Tidak relevan
77. Lainnya …….

F2. Pemanfaatan posyandu					
4	Pernahkah ibu pergi ke posyandu untuk memeriksakan kesehatan dan penimbangan anak?	0. Tidak (lompat ke no.8)<br>1. Ya<br>66. Tidak relevan<br>88. tidak tahu<br>99. tidak menjawab		[ ]	[UtilizeHS]
5	Dalam 3 bulan terakhir, berapa kali anda mengunjungi posyandu?	1. 1 kali<br>2. 2 kali<br>3. 3 kali<br>66. tidak relevan jika, tidak mengunjungi posyandu<br>88. tidak tahu<br>99. tidak menjawab		[ ]	[freqHS]
6	Jika ya, pelayanan apa saja yang diterima di posyandu? **(BACAKAN JAWABANNYA)** **(JAWABAN BISA LEBIH DARI SATU)**	a. Penimbangan berat badan anak	Kode:<br>0. Tidak<br>1. Ya<br>66. tidak relevan, jika tidak mengunjingi posyandu<br>88. tidak tahu	a. [ ]	[F5a]
		b. Pengukuran tinggi badan anak		b. [ ]	[F5b]
		c. Imunisasi anak		c. [ ]	[F5c]
		d. Penyuluhan gizi & kesehatan		d. [ ]	[F5d]
		f.Pengobatan ringan anak (oralit,obat cacing, dll)		f. [ ]	[F5f]
		g. PMT (pemberian makanan tambahan) anak		g. [ ]	[F5g]
		h. Suplementasi vitamin A anak		h. [ ]	[F5h]

Kecamatan	Desa	No. Responden
[ ][ ]	[ ][ ]	[ ][ ][ ]

7	Siapa yang memberikan informasi kesehatan/gizi di posyandu? **(JAWABAN BISA LEBIH DARI SATU)**	a. Petugas gizi	Kode:<br>0. Tidak<br>1. Ya<br>66. Tidak relevan<br>77. lainnya<br>88. tidak tahu	a.[ ]	[F6a]
		b.Kader		b.[ ]	[F6b]
		c.Bidan		c.[ ]	[F6c]
		d.Perawat/mantri		d.[ ]	[F6d]
		e. lainnya……………		e.[ ]	[F6e]
**F3. Status Imunisasi**					
8	Apakah anak anda pernah diimunisasi?	1. Ya → **lanjut ke no 10**<br>0. Tidak<br>66. Tidak relevan<br>88. Tidak tahu<br>99. Tidak menjawab		[ ]	[ImmuStat]
9	Mengapa anak anda tidak pernah diimunisasi?<br><br>**(JAWABAN HANYA BOLEH 1)**	1. Tidak diizinkan keluarga<br>2. Takut anak demam<br>3. Anak sering sakit (sakit pada periode pemberian vaksin sehingga ditunda/tidak boleh)<br>4. Tidak tahu tempat imunisasi<br>5. Tempat imunisasi terlalu jauh<br>66. Tidak relevan<br>77. Lainnya, …………..<br>88. Tidak tahu<br>99. Tidak menjawab		[ ]	[ImmuRsn]<br>[ImmuLainnya]
10	Imunisasi apa saja yang sudah diterima anak?<br><br>Kode:<br>**0. Tidak**<br>**1. Ya**<br>**66. Tidak relevan (untuk anak yang tidak diimunisasi/ belum cukup umur)**<br>**88. Tidak tahu**	Jenis imunisasi			
		0-7 hari	a. HB0	[ ]	[typeImmu1]
		1 bulan	b. BCG	[ ]	[typeImmu2]
			c. Polio 1	[ ]	[typeImmu3]
		2 bulan	d. DPT	[ ]	[typeImmu4]
			e. HB	[ ]	[typeImmu5]
			f. Hib 1	[ ]	[typeImmu6]
			g. Polio 2	[ ]	[typeImmu7]
		3 bulan	h. DPT	[ ]	[typeImmu8]
			i. HB	[ ]	[typeImmu9]
			j. Hib 2	[ ]	[typeImmu10]
			k. Polio 3	[ ]	[typeImmu11]
		9 bulan	l. Campak	[ ]	[typeImm12]
11.	Sumber informasi imunisasi anak	1.Interview dengan responden<br>2. Buku KIA/KMS<br>3. Tenaga kesehatan (bidan, kader,dll)<br>66. tidak relevan jika anak tidak diimunisasi<br>77. Lainnya, …………		[ ]	[ImmuInfo]
**F4. Suplementasi vitamin A**					
12	Apakah [nama anak] pernah menerima kapsul vitamin A?<br><br>**(probing: kapsul biru, untuk anak 6-11 bulan)**	1. Ya<br>0. Tidak→ **lanjut ke G**<br>66. tidak relevan jika umur belum cukup<br>88. Tidak tahu<br>99. Tidak menjawab		[ ]	[VitARcv]
13	Siapa yang memberikan kapsul vitamin A tersebut?<br>(siapa **NAMA PETUGAS** yang memberikan)	1. Kader<br>2. Bidan<br>3. Perawat<br>66. tidak relevan jika tidak mendapat vitamin A<br>77. lainnya, …………..<br>88. tidak tahu<br>99. tidak menjawab		[ ]	[VitAAccpt]

Kecamatan		Desa		No. Responden		

14	Siapa yang meneteskan isi kapsul vitamin A ke anak?	1. Kader<br>2. bidan<br>3. perawat<br>4. ibu<br>5. pengasuh<br>66. tidak relevan jika tidak mendapat vitamin A<br>77. lainnya, ____________<br>88. tidak tahu<br>99. Tidak menjawab	[ ]	[VitAConsm]

G. Riwayat Penyakit Anak					
No	Pertanyaan		Kategori	Kode	
**G1. DIARE**					
1	Dalam 2 minggu terakhir, **<u>paling banyak</u>,** berapa kali **[nama anak]** BAB dalam sehari?		0. <3 kali<br>1.≥ 3kali	[ ]	[diareFreq]
2	Bagaimana konsistensinya?		0. Tidak encer/cair<br>1. Encer/cair	[ ]	[diareCons]
**G2. INFEKSI SALURAN PERNAPASAN AKUT**					
3	Apakah **[nama anak]** pernah mengalami panas/ demam dalam 2 minggu terakhir?		0. Tidak → **lanjut ke H**<br>1.Ya	[ ]	[ARI]
4	Apakah disertai satu atau lebih gejala:<br><br>**BOLEH DIJAWAB LEBIH DARI SATU NAMUN KODE TETAP 1**	1. Batuk<br>2. Sakit tenggorokan<br>3. Pilek<br>4. Sesak nafas/ kesulitan bernafas	0. Tidak<br>1.Ya<br>66.tidak relevan jika tidak demam<br>88. tidak tahu	[ ]	[ARISymp]

**H. Air, Sanitasi dan Higiene**			Kode	
**H1. AIR**				
1a	Apa jenis sumber air utama untuk kebutuhan minum dan memasak yang **<u>paling sering digunakan</u>**?<br>(yang masuk ke dalam tubuh)	1. Air ledeng/PDAM<br>2. Air ledeng eceran/membeli<br>3. Sumur bor/pompa<br>4. Sumur gali terindung<br>5. Sumur gali tak terlindung<br>6. Mata air terindung<br>7. Mata air tidak terindung<br>8. Penampungan air hujan<br>9. Air sungai/danau/irigasi<br>10. Air kemasan<br>77. Lainnya:	[ ]	[DWsource]
1b	Apa jenis sumber air untuk kebutuhan lainnya seperti mencuci tangan, mencuci baju, dsb yang **<u>paling sering digunakan</u>**?	1. Air ledeng/PDAM<br>2. Air ledeng eceran/membeli<br>3. Sumur bor/pompa<br>4. Sumur gali terindung<br>5. Sumur gali tak terlindung<br>6. Mata air terindung<br>7. Mata air tidak terindung<br>8. Penampungan air hujan<br>9. Air sungai/danau/irigasi<br>77. Lainnya:	[ ]	[WaterSources]
2	Dimana letak sumber air untuk kebutuhan minum yang **<u>paling sering digunakan</u>**?	1. Dalam rumah→ **lanjut ke 4a**<br>2. Dalam pekarangan rumah → **lanjut ke 4a**<br>3. Diluar pekarangan rumah<br>77. Lainnya:	[ ]	[DWplace]
3	Berapa waktu yang diperlukan **<u>biasanya</u>** untuk memperoleh air kebutuhan minum? (total dari berangkat hingga kembali, termasuk menunggu)<br>**Prioritaskan waktu tempuh dengan berjalan kaki**	1. < 6 menit<br>2. 6-30 menit<br>3. 31-60 menit<br>4. >60 menit<br>66. Tidak Relevan, jika sumber air dari rumah	[ ]	[DWtime]
4a	Apakah anda melakukan pengolahan air minum sebelum diminum?	1. Ya<br>0. Tidak → **lanjut ke 4c**<br>88. Tidak tahu	[ ]	[DWsafe]

Kecamatan	Desa	No. Responden
[ ][ ]	[ ][ ]	[ ][ ][ ]

4b	Bagaimana cara pengolahan air sebelum diminum/ konsumsi oleh rumah tangga yang **paling sering digunakan**? (yang paling mendekati sebelum dikonsumsi)	1. Dengan pemanasan/dimasak<br>2. Dengan penyinaran matahari/UV<br>3. Ditambah larutan tawas/klorin<br>4. Disaring dan ditambah larutan tawas/klorin<br>5. Disaring/filtrasi saja<br>66. tidak relevan, jika jawaban 4a = tidak<br>77. Lainnya:	[ ]	[DWprocess]
4c	Apa jenis sarana/tempat penyimpanan air siap minum **yang paling sering** digunakan?	1. Dispenser<br>2. Teko/ceret/termos/jerigen<br>3. Kendi/periuk/tempayan<br>4. Ember/panci tertutup<br>5. Ember/panci terbuka<br>77. Lainnya:	[ ]	[DWstorage]
**H2. SANITASI**				
5a	Penggunaan fasilitas tempat buang air besar sebagian besar anggota rumah tangga **yang biasa digunakan**?	1. Milik sendiri<br>2. Milik bersama<br>3. Umum<br>4. Tidak ada→ **lanjut ke 5c**	[ ]	[toiletHH]
5b	Apa jenis kloset **yang biasa digunakan**?	1. Leher angsa<br>2. Plengsengan<br>3.Cemplung/ Cubluk<br>77.Lainnya:..............<br>66. Tidak relevan, jika tidak menggunakan fasilitas BAB	[ ]	[toilettype]
5c	Apa tempat pembuangan akhir tinja **yang biasa digunakan**?	1. Septik Tank<br>2. SPAL (Saluran Pembuangan Air Limbah)<br>3. Kolam/sawah<br>4. Sungai/danau/laut<br>5. Lubang tanah<br>6. Pantai/tanah lapang/kebun<br>77. Lainnya:.................<br>88. Tidak tahu<br>99. Tidak menjawab	[ ]	[fcs5c]
6	Saat **terakhir kali** anak (dibawah umur 1 tahun) di rumah anda BAB, apa yang dilakukan untuk membuang tinja nya?<br><br>**[TIDAK MEMBACAKAN PILIHAN JAWABAN]**<br>**Utamakan target anak**	1. Anak BAB di Toilet<br>2. Dibilas ke dalam toilet<br>3. Dibilas ke tempat pembuangan<br>4. Dibuang ke tempat sampah<br>5. Dikubur<br>6. Dibiarkan terbuka/ dibuang sembarangan<br>77. Lainnya:.................	[ ]	[fcs6]
7	Apa jenis tempat pengumpulan/ penampungan sampah basah (organik/bekas makanan) **yang biasa digunakan** di dalam rumah?	1. Tempat sampah tertutup<br>2. Tempat sampah terbuka<br>3. Tidak ada tempat sampah	[ ]	[trshTyp]
8	Bagaimana cara penanganan sampah rumah tangga **yang biasa dilakukan**?	1. Diangkut petugas/dibuang langsung ke TPA<br>2. Ditimbun dalam tanah<br>3. Dibuat kompos<br>4. Dibakar<br>5. Dibuang ke kali/ parit/ laut<br>6. Dibuang sembarangan<br>7. diberikan ke hewan<br>77. lainnya, ……….	[ ]	[trshHandlng]
9	Dimana tempat pembuangan air limbah dari bukan kloset **yang biasa digunakan**?	1. Penampungan tertutup di pekarangan/ SPAL<br>2. Penampungan terbuka di pekarangan<br>3. Penampungan di luar pekarangan<br>4. Tanpa penampungan (di tanah)<br>5. Langsung ke got/ sungai→ **lanjut ke H3**	[ ]	[wastePlc]
10	Bagaimana kepemilikan sarana pembuangan air limbah dari kamar mandi /dapur/ tempat cuci **yang biasa digunakan**?	1. Sendiri/ rumahtangga<br>2. Bersama/ komunal<br>66. Tidak Relevan, jika dibuang langsung ke got/sungai	[ ]	[wasteH10]
**H3. PRAKTIK HIGIENE**				
11	Apakah [RUMAH TANGGA] memiliki tempat untuk cuci tangan selain dikamar mandi?	1. Ya<br>0. Tidak→ **lanjut ke no 13**	[ ]	[HWfaclts]

Kecamatan		Desa		No. Responden		

12	Dimana biasanya setiap anggota [RUMAH TANGGA] mencuci tangan di rumah<br>**66. tidak relevan, jika jawaban no 11=tidak**				[HWfacltsTyp]<br><string>
13	Kapan ibu mencuci tangan ibu **<u>yang terakhir kali</u>?**<br><br>**[TIDAK MEMBACAKAN PILIHAN JAWABAN DAN JAWABAN HANYA 1]**	1. Sebelum makan<br>2. Sebelum menyiapkan makanan<br>3. Sebelum menyuapi anak makan<br>4. Setelah menggunakan toilet<br>5. Setelah mengganti popok/pembalut<br>6. Setelah makan<br>7. Sebelum dan setelah mengobati luka<br>8. Setelah membersihkan dan membuang sampah<br>9. Setelah bersentuhan dengan hewan/kotoran hewan<br>10. Setelah menyentuh orang sakit<br>11. Setelah bepergian<br>12. Setelah bersentuhan dengan hewan<br>77. Lainnya:.............<br>88. Tidak ingat		[ ]	[HygPract]
14	Bagaimana cara ibu mencuci tangan yang **<u>terakhir kali</u>?**<br><br>**[TIDAK MEMBACAKAN PILIHAN JAWABAN DAN MINTA IBU MENJELASKAN CARA IBU MENCUCI TANGAN SECARA RINCI]**	1. Membasuh tangan dengan air saja di dalam wadah (air tidak mengalir)<br>2. Membasuh tangan dengan air mengalir<br>3. Membasuh tangan dengan sabun dan air di dalam wadah<br>4. Membasuh tangan dengan sabun dan air bersih mengalir<br>77. Lainnya:		[ ]	[HygLatest]
15	**<u>Kapan saja</u>** biasanya ibu mencuci tangan?<br><br>**TIDAK MEMBACAKAN PILIHAN JAWABAN**<br><br>**[JAWABAN BOLEH LEBIH DARI 1]**<br><br>**PROBING: APA LAGI?**	a. Setiap kali tangan kotor	1. Ya<br>0. Tidak<br>77. Lainnya<br>88. Tidak tahu	[ ]	[HygTime1]
		b. Setelah BAB		[ ]	[HygTime2]
		c. Setelah mengganti popok/pembalut		[ ]	[HygTime3]
		d. Sebelum makan		[ ]	[HygTime4]
		e. Sebelum member makan anak		[ ]	[HygTime5]
		f. Sebelum memegang makanan		[ ]	[HygTime6]
		g. Sebelum menyusui bayi		[ ]	[HygTime7]
		h. Lainnya:.............		[ ]	[HygTime8]
16a	**<u>Observasi kuku tangan pengasuh saat wawancara</u>**	1. Kuku kotor, kuku panjang<br>2. Kuku kotor, kuku pendek<br>3. Kuku bersih, kuku panjang<br>4. Kuku bersih, kuku pendek		[ ]	[CGnails]
16b	**<u>Observasi kuku tangan anak saat wawancara</u>**	1. Kuku kotor, kuku panjang<br>2. Kuku kotor, kuku pendek<br>3. Kuku bersih, kuku panjang<br>4. Kuku bersih, kuku pendek<br>66. Tidak relevan jika anak tidak hadir		[ ]	[CHnails]

**I. Paparan Informasi Umum dan Kesehatan**			**Kode**	
1	Seberapa sering Ibu membaca majalah atau koran?<br>(tidak relevan untuk yang buta aksara)	1. Selalu (Setiap hari)<br>2. Sering (3-4 kali/minggu)<br>3. Kadang- kadang<br>4. Tidak pernah	[ ]	[I1]
2	Seberapa sering Ibu mendengarkan radio?	1. Selalu (Setiap hari)<br>2. Sering (3-4 kali/minggu)<br>3. Kadang- kadang<br>4. Tidak pernah	[ ]	[I2]
3	Seberapa sering Ibu menonton televisi?	1. Selalu (Setiap hari)<br>2. Sering (3-4 kali/minggu)<br>3. Kadang- kadang<br>4. Tidak pernah	[ ]	[I3]

Kecamatan		Desa		No. Responden		

4.	Seberapa sering ibu mengakses internet?	1. Selalu (Setiap hari)<br>2. Sering (3-4 kali/minggu)<br>3. Kadang- kadang<br>4. Tidak pernah		[ ]	[I4]
5	Apakah Ibu pernah mendapatkan informasi tentang kesehatan?	0. Tidak → lanjut ke no. 8<br>1. Ya		[ ]	[I5]
6	Jika ya, dari mana biasanya Ibu mendapatkan informasi tentang kesehatan?<br>**JAWABAN TIDAK DIBACAKAN**<br>**JAWABAN BISA LEBIH DARI SATU**<br>**PROBING: APA LAGI?**	a. Televisi	0. Tidak<br>1. Ya<br>77. lainnya<br>66. tidak relevan jika tidak pernah mendapat informasi	[ ]	[I6]
		b.Tenaga kesehatan		[ ]	
		c. Kader		[ ]	
		d. Keluarga/teman		[ ]	
		e. Radio		[ ]	
		f. Koran/majalah		[ ]	
		g. internet		[ ]	
		h. Lainnya, Sebutkan		[ ]	
7	Informasi kesehatan apa saja yang pernah ibu dapatkan?	a. Gizi	0. Tidak<br>1. Ya<br>66. tidak relevan<br>77. lainnya	[ ]	[HIcontent]
		b. Penanggulangan diare		[ ]	
		c. Pengobatan ringan anak (contoh : oralit)		[ ]	
		d. Suplementasi anak		[ ]	
		e. Imunisasi anak		[ ]	
		f. Lainnya_______		[ ]	
8	Apakah Ibu memiliki buku KIA?	1. Ya<br>0. Tidak → **lanjut ke J**		[ ]	[KIAposs]
9	Seberapa sering Ibu membaca buku KIA selama kehamilan?<br>66. tidak relevan	1. Selalu (Setiap hari)<br>2. Sering (3-4 kali/minggu)<br>3. Kadang- kadang (atau Cuma 1x baca)<br>4. Tidak pernah (Lanjutkan ke no.9)		[ ]	[KIAfreq]
10	Informasi apa yang menarik ketika Ibu membaca buku KIA selama kehamilan?<br>66. tidak relevan				[KIAintrestng]
11.	Seberapa sering Ibu membaca buku KIA setelah melahirkan?<br>66. tidak relevan	1. Selalu (Setiap hari)<br>2. Sering (3-4 kali/minggu)<br>3. Kadang- kadang<br>4. Tidak pernah (lanjutkan ke J)		[ ]	[KIAfreqAftr]
11.	Informasi apa yang menarik ketika Ibu membaca buku KIA setelah melahirkan?<br>66. tidak relevan				[KIAintrestngAftr]

**J. PRAKTIK PEMBERIAN MAKAN PADA ANAK**				
**J1. STATUS MENYUSUI**				
1	Apakah ibu kandung pernah menyusui [nama anak]?	0. Tidak Pernah<br>1. Pernah → **lanjut ke 3**<br>88. Tidak Tahu	[ ]	[BF]
2	Mengapa **biasanya** [nama ibu] tidak Menyusui bayi?<br><br>**LANJUT KE NO 7**	1. Saya memliki penyakit tertentu/saya sakit<br>2. ASI tidak keluar<br>3. Tidak dizinikan suami<br>4. Mempengaruhi bentuk payudara<br>5. Sibuk/bekerja jauh dari rumah<br>66. Tidak relevan, jika ibu menyusui anaknya<br>77. Lainnya, Sebutkan__________<br>99. Tidak Menjawab<br>88. Tidak Tahu	[ ]	[notBFRsn]
**J2. INISASI MENYUSUI DINI**				
3	Kapan ibu (kandung) pertama kali memberikan ASI pada bayi?<br>**CATAT JAM/HARI YANG DISEBUTKAN OLEH RESPONDEN**	1. Segera (≤ 1 jam)<br>2. 1-24 jam<br>3. ≥ 24 jam<br>66. Tidak relevan jika tidak ASI<br>88. Tidak tahu<br>99 Tidak Menjawab	[ ]	[EBI]

Kecamatan	Desa	No. Responden
[ ][ ]	[ ][ ]	[ ][ ][ ]

4	Bagaimana cara ibu kandung memberikan ASI pertama kali kepada bayi?	1. Bayi di dada ibu dengan pakaian<br>2. Setelah lahir bayi langsung diletakkan diatas dada ibu dan membiarkan bayi mencari putting susu ibu<br>66. Tidak relevan jika tidak ASI<br>77. lainnya, Sebutkan__________<br>88. Tidak tahu<br>99 Tidak Menjawab		[ ]	[EBIHow]
**J3. ASI EKSKLUSIF**					
5	Pada 3 (tiga) hari pertama setelah melahirkan (sebelum ASI keluar dengan lancar) apakah bayi diberikan makanan dan minuman selain ASI? (prelakteal)	0. Tidak → **lanjut ke no. 7**<br>1. Ya<br>66. Tidak relevan jika tidak ASI<br>88. Tidak Tahu		[ ]	[EBF]
6	Makanan/minuman apa yag diberikan kepada bayi saat itu?<br><br>**CATAT SEMUA YANG DISEBUTKAN**<br><br>**PILIHAN BOLEH DIBACAKAN**<br><br>**JAWABAN BOLEH LEBIH DARI SATU**	a. Susu formula	0. Tidak<br>1. Ya<br>66. Tidak relevan jika tidak ASI sama sekali<br>77. lainnya<br>88. Tidak tahu	[ ]	[nEBFftyp1]
		b. Susu yang lain		[ ]	[nEBFftyp2]
		c. Air putih		[ ]	[nEBFftyp3]
		d. Air gula		[ ]	[nEBFftyp4]
		e. Air tajin (rebusan beras)		[ ]	[nEBFftyp5]
		f. Sari buah/jus buah		[ ]	[nEBFftyp6]
		g. teh		[ ]	[nEBFftyp7]
		h. madu		[ ]	[nEBFftyp8]
		i. Makanan lunak		[ ]	[nEBFftyp9]
		j. lainnya, sebutkan____________________		[ ]	[nEBFftyp10]
7	Pada usia berapa anak anda diberikan makanan/minuman selain ASI?<br>**TANYAKAN LAGI APAKAH SEBELUMNYA ADA DIBERIKAN SESUATU.**<br><br>(pembulatan ke bawah → misal: 5 bulan 3 minggu → dianggap 5 bulan. Karena artinya sudah diberi makanan sebelum 6 bulan.<br>Baru 3 minggu atau pernah diberi makanan prelakteal → dianggap 0 bulan)	_______ bulan<br>66. Tidak Relevan, jika bayi diberikan ASI saja<br>88. Tidak tahu<br>99. Tidak menjawab		[ ]	[nEBFage]
8	Makanan/minuman selain ASI apa **yang pertama kali** diberikan pada usia tersebut (usia pada no 7)?	1. susu formula<br>2. susu yang lain<br>3. air putih<br>4. air gula<br>5. tajin<br>6. sari buah/jus<br>7. teh	8. madu<br>9. makanan lunak<br>10. lainnya, sebutkan<br>66. Tidak relevan jika tidak ASI<br>88. Tidak tahu<br>99. Tidak jawab	[ ]	[nEBFftyp]
9	Mengapa anda memberikan makanan/minuman selain ASI pada usia tersebut (usia pada no 7)?<br><br>**BOLEH LEBIH DARI 1**<br><br>**PILIHAN TIDAK DIBACAKAN**	a. Anak masih terlihat lapar	0. Tidak<br>1. Ya<br>66. tidak relevan jika hanya ASI saja<br>77. lainnya<br>88. Tidak Tahu	[ ]	[nEBFrsn1]
		b. Anak menangis		[ ]	[nEBFrsn2]
		c. ASI tidak cukup		[ ]	[nEBFrsn3]
		d. Berdasarkan rekomendasi dari ....______		[ ]	[nEBFrsn4]
		e. Saat yang tepat memberikan makanan/minuman yang lain		[ ]	[nEBFrsn5]
		f. Ibu harus bekerja		[ ]	[nEBFrsn6]
		g. Lainnya, Sebutkan		[ ]	[nEBFrsn7]

Kecamatan	Desa	No. Responden
[ ][ ]	[ ][ ]	[ ][ ][ ]

J4. MAKANAN PENDAMPING ASI (hanya untuk yang masih mendapatkan ASI)				
10	Apakah **kemarin** anak ibu diberikan makan lunak atau padat pada?	0. Tidak, **(lanjut ke no.13)**<br>1. Ya<br>66. Tidak Relevan, jika bayi diberikan ASI saja atau tidak minum ASI sama sekali<br>88. Tidak tahu	[ ]	[CF]
11	Sebutkan jenis MP ASI yang **pertama kali** diberikan kepada bayi?	1. Makanan Lunak<br>2. Makanan padat<br>66. Tidak Relevan, jika bayi diberikan ASI saja atau tidak minum ASI sama sekali<br>88. Tidak tahu	[ ]	[CFfirst]
12	Berapa kali **kemarin** anak ibu diberi MP ASI?	Jumlah frekuensi___________kali<br>66. Tidak Relevan, jika bayi diberikan ASI saja<br>88. Tidak tahu	[ ]	[CFfreq]
**J5. MINUM DENGAN BOTOL (hanya untuk yang masih mendapatkan ASI)**				
13	Apakah **kemarin** anak meminum ASI menggunakan sendok, mangkok atau botol?	0. Tidak<br>1. Ya<br>88 Tidak tahu<br>66. Tidak Relevan, bayi sudah tidak diberikan ASI	[ ]	[nEBF24hr]

K. PENGETAHUAN PENGASUH TENTANG MAKANAN, NUTRISI, DAN KESEHATAN

Kode:
0 = Jawaban responden berbeda dengan kunci jawaban
1 = Jawaban responden sama dengan kunci jawaban
88 = Tidak Tahu
99 = Tidak Jawab

B : Benar **S: Salah** **TT: Tidak Tahu** **TJ: Tidak Jawab**

No	Pernyataan	Jawaban Responden				Kunci	KODE	
		B	S	TT	TJ			
1	Tangan kita dapat menjadi penyebab terjadinya diare					B	[ ]	[CGknow1]
2	Anda tidak perlu mencuci tangan sebelum makan, jika Anda makan menggunakan sendok					S	[ ]	[CGknow2]
3	Diare berulang dapat menyebabkan kematian pada bayi					B	[ ]	[CGknow3]
4	Cara mencuci tangan yang benar adalah dengan menggunakan sabun dan air bersih yang mengalir					B	[ ]	[CGknow4]
5	Bayi berumur 0-6 bulan menyusu atau diberi makan dengan ASI sesuai kebutuhan, kapanpun bayinya meminta					B	[ ]	[Cgknow5]
6	Tekstur makanan pendamping ASI bagi bayi berumur dibawah 12 bulan dapat dibuat seperti makanan dewasa					S	[ ]	[Cgknow6]
7	Anak stunting/pendek bukanlah masalah gizi					S	[ ]	[Cgknow7]
8	Anak stunting/pendek terjadi jika tinggi badan anak lebih rendah dari standar berat badannya					S	[ ]	[Cgknow8]
9	Anak yang mengonsumsi makanan yang beragam dan bergizi seimbang akan meningkatkan resiko stunting/pendek					S	[ ]	[Cgknow9]
10	Dampak anak stunting/ pendek ialah penyakit di masa dewasa seperti penyakit jantung					B	[ ]	[CGknow10]
11	Anemia disebabkan kurang mengonsumsi zat besi					B	[ ]	[CGknow11]
12	Kehilangan nafsu makan, mual dan muntah merupakan tanda atau gejala anemia					B	[ ]	[CGknow12]
13	Mengonsumsi banyak sumber hewani dapat mencegah anemia					B	[ ]	[CGknow13]
14	Dampak anemia bagi anak-anak ialah keterlambatan pertumbuhan mental dan fisik					B	[ ]	[Cgknow14]

Kecamatan		Desa		No. Responden		

M. INFORMASI EKONOMI			Kode
**Pendapatan Rumah Tangga**			
1	Berapa jumlah anggota rumah tangga yang berkontribusi terhadap pendapatan keluarga?		[M1]
2	(see table below)		[M2a]/1<br>[M2b]/1<br>[M2c]/1<br>[M2d]/1<br>[M2e]/1<br>[M2f]/1<br>[M2g]/1<br>[M2h]/1<br>[M2i]/1<br>[M2j]/1<br>[M2t]

Rata-rata Pendapatan Keluarga Selama 1 bulan terakhir*

No	Sumber Pendapatan (orangnya)	Jumlah Penghasilan		
		Harian	Mingguan	Bulanan
a				
b				
c				
d				
e				
f				
g				
h				
i				
j				
**TOTAL [**M2t]				………………………

***recall pendapatan dilakukan 3 bulan terakhir untuk mengantisipasi responden yang pendapatannya tidak setiap bulan**

Pengeluaran Rumah Tangga

3 Berapa banyak biasanya pengeluaran rumah tangga dalam **1 bulan terakhir**?
Catatan : khusus untuk pengeluaran makanan, ditanyakan dalam 1 minggu terakhir

Variabel	Jumlah (Rupiah)				Total sebulan	Kode
	Harian	Mingguan	Bulanan	Tahunan		
a. Makanan (nasi, mie, minyak goreng, susu, kopi, gula, telur, sayur, buah, teh, dsb) **(untuk hitungan-hitungan item satu-persatu, dihitung di halaman sebaliknya. Isi kolom ini BERSIH TOTAL MAKANAN**)						[M3a]
b. Kesehatan						[M3b]
c. Pendidikan (SPP)						[M3c]
d. Tagihan rumah tangga :						[M3d]
1. Listrik						[M3d1]
2. Air						[M3d2]
3. Telepon						[M3d3]

Kecamatan	Desa	No. Responden

4. Asuransi						[M3d4]
5. Pajak						[M3d5]
6.Bahan rumah tangga (sabun, sampo, odol, tisu, dll) **(untuk hitungan-hitungan item satu-persatu, dihitung di halaman sebaliknya. Isi kolom ini BERSIH TOTAL BAHAN RUMAH TANGGA**)						[M3d6]
7. Popok						[M3d7]
8. Pulsa						[M3d8]
9. Elpiji						[M39]
e. Transportasi (bensin, kendaraan umum)						[M3e]
f. Aktivitas sosial (iuran rutin, arisan)						[M3f]
g. Rokok						[M3g]
h. Jajan anak						[M3h]
i. Tabungan						[M3i]
j. Kebutuhan lain-lain (pupuk, benih, perawatan alat pertanian/perahu dsb)						[M3j]
**Total**						[M3t]

Kecamatan	Desa	No. Responden
[ ] [ ]	[ ] [ ]	[ ] [ ] [ ]

**N. KETAHANAN PANGAN RUMAH TANGGA**[FS]<br>***<u>*untuk setiap pertanyaan yang diloncat, jawaban diisi dengan 66. Tidak relevan</u>***				
**Pertanyaan kecukupan pangan pilihan/penyaring: PERTANYAAN 1**<br>Pertanyaan ini tidak digunakan untuk mengukur kecukupan pangan/ skala kelaparan. Pertanyaan ini dapat digunakan dalam kaitannya dengan pendapatan, sebagai penyaring awal untuk mengurangi beban responden dengan pendapatan tinggi.				
1.	Saya akan membacakan beberapa kalimat, menurut ibu manakah kalimat yang menggambarkan keadaan makanan dirumah ibu, **<u>dalam 12 bulan terakhir</u>?**	1. Jumlahnya cukup dan jenisnya sesuai dengan yang ingin keluarga ibu makan<br>2. Jumlahnya cukup, tetapi bukan selalu jenis makanan yang ingin keluarga ibu makan<br>3. Kadang-kadang jumlah yang dimakan tidak cukup<br>4. Sering tidak cukup<br>88. Tidak tahu<br>99. Tidak menjawab	[ ]	[FS1]
**<u>TAHAP RUMAH TANGGA 1 (pertanyaan 2 – 4)</u>**<br>Sekarang saya akan membacakan pada ibu beberapa pernyataan yang dikemukakan orang tentang situasi makanan. Untuk setiap pernyataan tolong beritahu saya apakah keadaan itu **<u>benar, kadang benar</u>** <u>atau</u> **<u>tidak benar</u>** dengan keadaan keluarga ibu selama **<u>12 bulan terakhir</u>**, sejak April tahun lalu.				
2.	"**Ibu <u>cemas</u> persediaan makanan sudah habis sebelum ibu punya uang untuk membeli lagi**". Dalam 12 bulan terakhir, apakah pernyataan tersebut sering benar, kadang benar, atau tidak benar bagi **keluarga** ibu?	1. Sering benar<br>2. Kadang benar<br>3. Tidak benar<br>88. Tidak tahu<br>99. Tidak menjawab	[ ]	[FS2]
3.	"Makanan yang ibu beli **<u>sudah habis</u>**, dan ibu tidak punya uang untuk membeli lagi". Dalam 12 bulan terakhir, apakah pernyataan tersebut sering benar, kadang benar, atau tidak benar bagi **keluarga** ibu?	1. Sering benar<br>2. Kadang benar<br>3. Tidak benar<br>88. Tidak tahu<br>99. Tidak menjawab	[ ]	[FS3]
4.	"Ibu tidak sanggup mengupayakan makan makanan yang seimbang (nasi dengan sayur dan lauk/pauk) untuk keluarga". Dalam 12 bulan terakhir, apakah pernyataan tersebut sering benar, kadang benar, atau tidak benar bagi **keluarga** ibu?	1. Sering benar<br>2. Kadang benar<br>3. Tidak benar<br>88. Tidak tahu<br>99. Tidak menjawab	[ ]	[FS4]
**<u>TAHAP DEWASA 2 (pertanyaan 5 – 8)</u>**<br>Penyaringan untuk tahap 2: JIKA ADA JAWABAN **MENG'IYA'KAN** (i.e. "sering benar" atau "kadang benar") untuk **SALAH SATU** pertanyaan **2 – 4 di TAHAP RUMAH TANGGA 1** ATAU **memberi tanggapan 3 atau 4. untuk PERTANYAAN 1**, **<u>isikan TAHAP DEWASA 2 ini</u>**; jika <u>tidak, lanjut ke</u> **TAHAP ANAK 4. Isikan "66. Tidak relevan" JIKA TAHAP INI DILOMPAT.**				
5.	Dalam 12 bulan terakhir, **apakah <u>ibu atau orang dewasa di rumah pernah mengurangi jumlah porsi makan atau tidak makan</u> karena tidak punya cukup uang untuk membeli makanan**?	1. Ya<br>0. Tidak → **lanjut ke 6**<br>88. Tidak tahu → **lanjut ke 6**<br>99. Tidak menjawab→ **lanjut ke 6**	[ ]	[FS5]
5a.	[JIKA **YA] Seberapa sering** hal ini terjadi – hampir setiap bulan, dalam beberapa bulan tapi tidak setiap bulan, atau hanya dalam 1 atau 2 bulan saja?	1. Hampir setiap bulan<br>2. Beberapa bulan, tidak setiap bulan<br>3. Hanya 1 atau 2 bulan saja<br>66. tidak relevan jika jawaban no 5 selain 'ya'<br>88. Tidak tahu<br>99. Tidak menjawab	[ ]	[FS5a]
6.	Dalam 12 bulan terakhir, **apakah <u>ibu pernah makan kurang dari porsi yang seharusnya</u> (menurut ibu) dimakan karena tidak punya uang untuk membeli makanan** ?	1. Ya<br>0. Tidak<br>88. Tidak tahu<br>99. Tidak menjawab	[ ]	[FS6]
7.	Dalam 12 bulan terakhir, **apakah <u>ibu pernah merasa lapar</u> tapi tidak makan karena ibu tidak punya uang untuk membeli makanan**?	1. Ya<br>0. Tidak<br>88. Tidak tahu<br>99. Tidak menjawab	[ ]	[FS7]
8.	Dalam 12 bulan terakhir, **apakah ibu mengalami <u>penurunan berat badan</u> akibat tidak punya uang untuk membeli makanan**?	1. Ya<br>0. Tidak<br>88. Tidak tahu<br>99. Tidak menjawab	[ ]	[FS8]

Kecamatan	Desa	No. Responden
[ ] [ ]	[ ] [ ]	[ ] [ ] [ ]

**TAHAP DEWASA 3 (pertanyaan 9 – 9a)**<br>Penyaringan untuk TAHAP DEWASA 3: JIKA ADA JAWABAN MENG'IYA'KAN untuk SALAH SATU pertanyaan 5 – 8, **isikan TAHAP DEWASA 3 ini**; jika tidak, lanjut ke **TAHAP ANAK 4**. **Isikan "66. Tidak relevan" JIKA TAHAP INI DILOMPAT**				
9.	Dalam 12 bulan terakhir, **apakah ibu atau orang dewasa lain di keluarga pernah tidak makan sehari penuh karena ketiadaan uang untuk membeli makanan**?	1. Ya<br>0. Tidak → lewati 9a<br>88. Tidak tahu→ lewati 9a<br>99. Tidak menjawab → lewati 9a	[ ]	[FS9]
9a.	[JIKA YA] Berapa sering hal ini terjadi – hampir setiap bulan, dalam beberapa bulan tapi tidak setiap bulan, atau hanya dalam 1 atau 2 bulan saja?	1. Hampir setiap bulan<br>2. Beberapa bulan, tidak setiap bulan<br>3. Hanya 1 atau 2 bulan saja<br>66. tidak relevan jika jawaban no 9 selain 'ya'<br>88. Tidak tahu<br>99. Tidak menjawab	[ ]	[FS9a]
**TAHAP ANAK 4 (pertanyaan 10 – 12)**<br>Sekarang saya akan membacakan pada ibu beberapa pernyataan yang dikemukakan orang tentang kondisi makanan yang terkait dengan anak yang ada di rumah ibu. Untuk setiap pernyataan tolong beritahu saya apakah keadaan itu **SERING benar, KADANG benar** atau **TIDAK benar untuk anak ibu/ anak yang tinggal di rumah ibu.**				
10.	"**Ibu hanya membeli beberapa jenis makanan yang *murah* untuk memberi makan anak-anak karena sering kehabisan uang untuk membeli makanan**". Dalam 12 bulan terakhir, apakah pernyataan tersebut sering benar, kadang benar, atau tidak benar bagi keluarga ibu?	1. Sering benar<br>2. Kadang benar<br>3. Tidak benar<br>88. Tidak tahu<br>99. Tidak menjawab	[ ]	[FS10]
11.	"**Ibu tidak bisa memberi makan anak ibudengan makanan yang seimbang(terdiri dari nasi, lauk pauk, sayur), karena ibu tidak sanggup untuk membelinya**". Dalam 12 bulan terakhir, apakah pernyataan tersebut sering benar, kadang benar, atau tidak benar bagi keluarga ibu?	1. Sering benar<br>2. Kadang benar<br>3. Tidak benar<br>88. Tidak tahu<br>99. Tidak menjawab	[ ]	[FS11]
12.	"**Anak ibu tidak bisa mendapat cukup makan karena ibu tidak mampu membeli cukup makanan"**. Dalam 12 bulan terakhir, apakah pernyataan tersebut sering benar, kadang benar, atau tidak benar bagi keluarga ibu?	1. Sering benar<br>2. Kadang benar<br>3. Tidak benar<br>88. Tidak tahu<br>99. Tidak menjawab	[ ]	[FS12]
**TAHAP ANAK 5 (pertanyaan 13 – 15)**<br>Penyaringan untuk TAHAP ANAK 5: JIKA ADA JAWABAN MENG'IYA'KAN (i.e. "sering benar" atau "kadang benar") untuk SALAH SATU pertanyaan 10 – 12, **ISIKAN TAHAP ANAK 5 ini**; jika tidak, **akhiri wawancara**.<br>**Isikan "66. Tidak relevan" JIKA TAHAP INI DILOMPAT**				
13.	Dalam 12 bulan terakhir, sejak April tahun lalu, **apakah ibu mengurangi porsi makan anak ibu karena ibu tidak punya cukup uang untuk membeli makanan** ?	1. Ya<br>0. Tidak<br>88. Tidak tahu<br>99. Tidak menjawab	[ ]	[FS13]
14.	Dalam 12 bulan terakhir, apakah **anak-anak** ibu **pernah melewatkan waktu makan** (misalnya makan menjadi satu kali sehari) **akibat tidak ada uang untuk membeli makanan**?	1. Ya<br>0. Tidak→ **lanjut ke 15**<br>88. Tidak tahu → **lanjut ke 15**<br>99. Tidak menjawab→ **lanjut ke 15**	[ ]	[FS14]
14a.	[JIKA **YA**] **Berapa sering** hal ini terjadi – hampir setiap bulan, dalam beberapa bulan tapi tidak setiap bulan, atau hanya dalam 1 atau 2 bulan saja?	1. Hampir setiap bulan<br>2. Beberapa bulan, tidak setiap bulan<br>3. Hanya 1 atau 2 bulan saja<br>66. tidak relevan jika jawaban no 14 selain 'ya'<br>88. Tidak tahu<br>99. Tidak menjawab	[ ]	[FS14a]
15.	Dalam 12 bulan terakhir, **pernahkah anak-anak ibu tidak makan sehari penuh karena tidak ada uang membeli makanan**?	1. Ya<br>0. Tidak<br>88. Tidak tahu<br>99. Tidak menjawab	[ ]	[FS15]

Kecamatan		Desa		No. Responden		

O. KETERSEDIAAN PANGAN RUMAH TANGGA [FA]				
1.	Apakah ada anggota keluarga Anda yang mengolah lahan atau bertani **dalam satu tahun terakhir**?<br>*Probing: lahan tidak harus milik sendiri; "mengolah atau bertanam di pekarangan?"*	1. Ya<br>0. Tidak →**Lanjut ke 10**	[ ]	[FA1]

		No	a. Jenis lahan	b. Luas	c. Kepemilikan<br>1. Milik sendiri<br>2. Milik orang lain/sewa	
2.	Jenis lahan apa yang Anda olah/tanam serta luasnya?<br><br>66. Tidak relevan jika tidak mengolah lahan/ bertani<br>88. tidak tahu<br><br>**usahakan dapatkan data dalam meter**	1	Pekarangan (di dalam pagar rumah)	……….m²	[ ]	[FA2a1] [FA2a2] [FA2a3] <ada string>
		2	Kebun (di luar pagar rumah)	……….m²	[ ]	[FA2b1] [FA2b2] [FA2b3]
		3	Lainnya. Sebutkan________	……….m²	[ ]	[FA2c1] [FA2c2] [FA2c3]

O1. SAYUR-SAYURAN YANG DITANAM [01]				
3.	Apakah keluarga Anda menanam/mengolah sayuran?	1. Ya<br>0. Tidak →**Lanjut ke 10**<br>66. Tidak relevan jika tidak mengolah lahan/ bertani	[ ]	[FA3]
4.	Apakah lahan Anda memproduksi sayuran sepanjang tahun?	1. Ya →**Lanjut ke 6**<br>0. Tidak<br>66. Tidak relevan jika tidak mengolah lahan/ bertani	[ ]	[FA4]
5.	Jika tidak sepanjang tahun, berapa bulan dalam setahun lahan Anda memproduksi sayuran?	………. bulan<br>66. Tidak relevan jika tidak mengolah lahan/ bertani	[ ]	[FA5]

6. Total jumlah jenis sayuran, produksi dan penjualan dari lahan dalam **2 bulan terakhir**
(Tulis jenis sayuran yang ditanam dan menghasilkan paling banyak)

No	a.Jenis sayuran	b.Produksi (kg)<br>[888. tidak tahu<br>999. belum panen]	c.Dijual<br>[0.Tidak 1. Ya 2. Belum panen<br>66. Tidak relevan jika tidak mengolah]
1			[ ]
2			[ ]
3			[ ]
4			[ ]
5			[ ]

[FA6a1] [FA6a2] [FA6a3] [FA6a4] [FA6a5] <string>
[FA6b1] [FA6b2] [FA6b3] [FA6b4] [FA6b5]
[FA6c1] [FA6c2] [FA6c3] [FA6c4] [FA6c5]

7.	Apa masalah utama lahan Anda?<br>***Jangan bacakan pilihan jawaban***<br>***Jika lebih dari 1, tanyakan prioritas***	a. Keterbatasan kemampuan/pengetahuan	0. Tidak<br>1. Ya<br>2. Prioritas *(hanya satu)*<br>66. Tidak relevan jika tidak mengolah lahan<br>88. tidak tahu	[ ]	[FA7a]
		b. Serangga/hama/penyakit		[ ]	[FA7b]
		c. Kekeringan/tidak ada hujan		[ ]	[FA7c]
		d. Kebanjiran		[ ]	[FA7d]
		e. Kekurangan benih/bibit		[ ]	[FA7e]
		f. Kekurangan tenaga kerja		[ ]	[FA7f]
		g. Tanaman tidak cocok di lahan tersebut		[ ]	[FA7g]
		h. Lainnya. Sebutkan: ……….		[ ]	[FA7h]
Jika keluarga atau ibu merasa tidak ada masalah, isi semua dengan 'tidak'					
8.	Darimana sumber benih/bibit Anda dalam 2 bulan terakhir?<br>***Bacakan pilihan jawaban***	a.Pribadi	1. Ya<br>0. Tidak<br>66. Tidak relevan jika tidak mengolah lahan<br>88. tidak tahu	[ ]	[FA8a]
		b.Pasar domestik		[ ]	[FA8b]
		c.Pemerintah		[ ]	[FA8c]
		d.Kelompok tani		[ ]	[FA8d]
		e.Tetangga/kerabat/sanak saudara		[ ]	[FA8e]
		f. Bantuan		[ ]	[FA8f]
		g.Lainnya. Sebutkan: ……….		[ ]	[FA8g]

Kecamatan	Desa	No. Responden
[ ][ ]	[ ][ ]	[ ][ ][ ]

9.	Seberapa besar porsi sayuran yang diproduksi dari lahan Anda yang dikonsumsi keluarga Anda dalam 2 bulan terakhir?	1. Seluruhnya<br>2. Tiga perempat<br>3. Setengah<br>4. Seperempat<br>5. Kurang dari seperempat<br>6. Tidak ada (belum panen)<br>66. Tidak relevan jika tidak mengolah lahan<br>88. tidak tahu	[ ]	[FA9]
10.	Darimana sumber utama sayuran yang dikonsumsi keluarga Anda dalam 2 bulan terakhir?	1. Produksi sendiri<br>2. Pasar domestik<br>3. Pemberian<br>66. Tidak relevan<br>77. Lainnya. Sebutkan: …………………	[ ]	[FA10]
11.	Apakah Anda membeli sayuran untuk keperluan keluarga Anda dalam 2 bulan terakhir?	1. Ya<br>0. Tidak →**Lanjut ke 13**	[ ]	[FA11]

12.	Jenis sayuran apa yang Anda beli dalam 2 bulan terakhir?<br>**BACAKAN PILIHAN JAWABAN**<br>**JAWABAN BISA LEBIH DARI SATU**	a. Sayuran berdaun hijau	1. Ya<br>0. Tidak<br>66. Tidak Relevan	[ ]	[FA12a]
		b. Sayuran dari akar (lobak, kentang, dll)		[ ]	[FA12b]
		c. Sayuran berwarna kuning/oranye		[ ]	[FA12c]
		d. Kacang-kacangan (kecipir, kacang merah, kacang panjang, kacang hijau, buncis, dll)		[ ]	[FA12d]
		e. Lainnya. Sebutkan: ……….		[ ]	[FA12e]

**O2. BUAH-BUAHAN YANG DITANAM** [02]				
13.	Apakah Anda memiliki pohon buah? (baik di pekarangan maupun di kebun)	1. Ya<br>0. Tidak →**Lanjut ke 17**	[ ]	[FA13]

14. Total jumlah jenis buah, produksi dan hasil penjualan **dalam 2 bulan terakhir**
(Tulis jenis buah yang ditanam dan **menghasilkan paling banyak**.)

No	a.Jenis buah	b.Produksi (kg)	c.Dijual [0.Tidak 1. Ya 2. Belum panen 66. Tidak relevan jika tidak mengolah]
1			[ ]
2			[ ]
3			[ ]
4			[ ]
5			[ ]
		Kode:<br>888. tidak tahu<br>999. belum panen	

[FA14a1] [FA14a2] [FA14a3] [FA14a4] [FA14a5] <string>
[FA14b1] [FA14b2] [FA14b3] [FA14b4] [FA14b5]
[FA14c1] [FA14c2] [FA14c3] [FA14c4] [FA14c5]

15.	Darimana sumber benih/bibit Anda dalam 2 bulan terakhir?<br>**BACAKAN PILIHAN JAWABAN**<br>**JAWABAN BISA LEBIH DARI SATU**	a.Pribadi	1. Ya<br>0. Tidak<br>66. Tidak relevan jika tidak memiliki pohon buah<br>88. Tidak tahu	[ ]	[FA15a]
		b.Pasar domestik		[ ]	[FA15b]
		c.Pemerintah		[ ]	[FA15c]
		d.Kelompok tani		[ ]	[FA15d]
		e.Tetangga/kerabat/sanak saudara		[ ]	[FA15e]
		f. Bantuan		[ ]	[FA15f]
		g.Lainnya. Sebutkan: ……….		[ ]	[FA15g]

16.	Seberapa besar porsi buah-buahan yang diproduksi dari lahan Anda yang dikonsumsi keluarga Anda dalam **2 bulan terakhir**?	1. Seluruhnya<br>2. Tiga perempat<br>3. Setengah<br>4. Seperempat<br>5. Kurang dari seperempat<br>6. Tidak ada (belum panen)<br>66. Tidak relevan jika tidak memiliki pohon buah<br>88. Tidak tahu	[ ]	[FA16]

Kecamatan	Desa	No. Responden
[ ][ ]	[ ][ ]	[ ][ ][ ]

17.	Darimana sumber utama buah-buahan yang dikonsumsi keluarga Anda dalam **2 bulan terakhir**?		1. Produksi sendiri<br>2. Pasar domestik<br>3. Pemberian<br>66. Tidak relevan jika tidak memiliki pohon buah<br>77. Lainnya. Sebutkan: ……….	[ ]	[FA17]
18.	Apakah Anda membeli buah-buahan untuk keperluan keluarga Anda dalam **2 bulan terakhir**?		1. Ya<br>0. Tidak →**Lanjut ke 20**	[ ]	[FA18]
19.	Jenis buah-buahan apa yang Anda beli dalam 2 bulan terakhir?<br>***BACAKAN PILIHAN JAWABAN***<br><br>***JAWABAN BISA LEBIH DARI SATU***	a. Beri (stroberi, dll)	1. Ya<br>0. Tidak<br>66. Tidak relevan jika tidak membeli buah	[ ]	[FA19a]
		b. Citrus (jeruk, lemon, limo, dll)		[ ]	[FA19b]
		c. Melon, semangka, dll		[ ]	[FA19c]
		d. Aprikot, ceri, persik, plum, dll		[ ]	[FA19d]
		e. Tropis (pisang, mangga, pepaya, nanas)		[ ]	[FA19e]
		f. Lainnya. Sebutkan: ……….		[ ]	[FA19f]
**O3. UNGGAS** [03]					
20.	Apakah keluarga Anda memiliki unggas dalam 2 bulan terakhir?	1. Ya<br>0. Tidak →**Lanjut ke 26**		[ ]	[FA20]
21.	Jika ya, **<u>berapa jumlah</u>** unggas yang Anda miliki dalam 2 bulan terakhir?	1. Ayam ……….<br>2. Bebek ……….<br>3. Burung dara ……….<br>4. Lainnya. Sebutkan: ……….<br>66. Tidak relevan jika tidak memiliki unggas		[ ]<br>[ ]<br>[ ]<br>[ ]	[FA211]<br>[FA212]<br>[FA213]<br>[FA214]
22.	Seberapa banyak konsumsi daging unggas yang berasal dari peliharaan Anda yang dikonsumsi oleh keluarga Anda dalam 2 bulan terakhir?	1. Seluruhnya<br>2. Tiga perempat<br>3. Setengah<br>4. Seperempat<br>5. Kurang dari seperempat<br>6. Tidak ada (belum panen)<br>7. Tidak dimakan (dipelihara dan sebagainya)<br>66. Tidak relevan jika tidak memiliki unggas		[ ]	[FA22]
23.	Dalam 2 bulan terakhir, seberapa banyak telur yang diproduksi unggas milik keluarga Anda?	………… Telur<br>66. Tidak relevan jika tidak memiliki telur atau tidak memiliki unggas<br>88. Tidak tahu		[ ]	[FA23]
24.	Seberapa banyak telur yang dikonsumsi oleh keluarga Anda yang berasal dari unggas (ayam) peliharaan keluarga Anda?	1. Seluruhnya<br>2. Tiga perempat<br>3. Setengah<br>4. Seperempat<br>5. Kurang dari seperempat<br>6. Tidak ada (belum panen)<br>7. tidak dimakan (ditetaskan dan sebagainya)<br>66. Tidak relevan jika tidak memiliki telur atau tidak memiliki unggas		[ ]	[FA24]
25.	Darimana sumber utama bibit unggas (ayam, bebek, burung dara, dll) Anda <u>yang diternak</u> dalam 2 bulan terakhir?<br>***BACAKAN PILIHAN JAWABAN***<br><br>***JAWABAN BISA LEBIH DARI SATU***	a.Pribadi	1. Ya<br>0. Tidak<br>66. Tidak relevan jika tidak memiliki ungags<br>88. tidak tahu	[ ]	[FA25a]
		b.Pasar domestik		[ ]	[FA25b]
		c.Pemerintah		[ ]	[FA25c]
		d.Peternakan lain		[ ]	[FA25d]
		e.Tetangga/kerabat/sanak saudara		[ ]	[FA25e]
		f. Bantuan		[ ]	[FA25f]
		g.Lainnya. Sebutkan: ……….		[ ]	[FA25g]

26.	Darimana sumber utama unggas (ayam, bebek, burung dara, dll) yang dikonsumsi keluarga Anda dalam 2 bulan terakhir?	1. Produksi sendiri<br>2. Pasar domestik<br>3. Pemberian<br>66. Tidak relevan tidak mengkonsumsi unggas<br>77. Lainnya. Sebutkan: ……….	[ ]	[FA26]
27.	Darimana sumber utama telur (ayam, bebek, burung dara, dll) yang dikonsumsi keluarga Anda dalam 2 bulan terakhir?	1. Produksi sendiri<br>2. Pasar domestik<br>3. Pemberian<br>66. Tidak relevan tidak mengkonsumsi telur<br>77. Lainnya. Sebutkan: ……….	[ ]	[FA27]
**O4. HEWAN TERNAK** [04]				
28.	Apakah keluarga Anda memiliki hewan ternak atau hewan yang digunakan untuk bertani dalam 2 bulan terakhir?	1. Ya<br>2. Tidak, tapi ada hewan titipan<br>3. Tidak punya dan tidak ada hewan titipan →**Lanjut ke 35**	[ ]	[FA28]
29.	*Jika jawaban 1. atau 2.* Berapa banyak hewan yang ada dalam 2 bulan terakhir?	1. ………. Kerbau<br>2. ………. Sapi<br>3. ………. Kambing, domba<br>4. ………. Lainnya. Sebutkan: ……….<br>66. Tidak relevan jika tidak memiliki hewan ternak	[ ]<br>[ ]<br>[ ]<br>[ ]	[FA301]<br>[FA302]<br>[FA303]<br>[FA304]
30.	Apakah ada hewan perah? (sapi, kambing, domba, kerbau) ***yang memproduksi susu***	1. Ya<br>0. Tidak →**Lanjut ke 33**<br>66. Tidak relevan jika tidak memiliki hewan ternak	[ ]	[FA30]
31.	Jika ya, berapa liter jumlah produksi susu per harinya?	………. Liter<br>66. Tidak relevan jika tidak ada hewan perah<br>88. Tidak tahu	[ ]	[FA31]
32.	Apa kegunaan utama dari susu yang diproduksi?	1. Dikonsumsi sendiri<br>2. Dijual<br>77. Lainnya. Sebutkan ……….<br>66. Tidak relevan jika tidak memproduksi susu	[ ]	[FA32]
33.	Berapa banyak daging yang dikonsumsi oleh keluarga Anda dalam 2 bulan terakhir yang merupakan hasil produksi ternak Anda sendiri?	1. Seluruhnya<br>2. Tiga perempat<br>3. Setengah<br>4. Seperempat<br>5. Kurang dari seperempat<br>6. Tidak ada (belum panen)<br>7. Tidak dimakan (dipelihara dan sebagainya)<br>66. Tidak relevan jika tidak memiliki hewan ternak dan/atau tidak mengkonsumsi daging	[ ]	[FA33]
34.	Darimana sumber utama bibit hewan yang diternak (kambing/domba, sapi/kerbau) Anda dalam 2 bulan terakhir? ***BACAKAN PILIHAN JAWABAN***<br><br>***JAWABAN BISA LEBIH DARI SATU***	a.Pribadi<br>b.Pasar domestik<br>c.Pemerintah<br>d.Peternakan lain<br>e.Tetangga/kerabat/sanak saudara<br>f. Bantuan<br>g.Lainnya. Sebutkan: ……….<br><br>1. Ya<br>0. Tidak<br>66. Tidak relevan jika tidak memiliki hewaan ternak<br>88. tidak tahu	[ ]<br>[ ]<br>[ ]<br>[ ]<br>[ ]<br>[ ]<br>[ ]	[FA34a]<br>[FA34b]<br>[FA34c]<br>[FA34d]<br>[FA34e]<br>[FA34f]<br>[FA34g]
35.	Darimana sumber utama daging hewan (kambing/domba, sapi/kerbau) yang dikonsumsi keluarga Anda dalam 2 bulan terakhir?	1. Produksi sendiri<br>2. Pasar domestik<br>3. Pemberian<br>66. Tidak relevan jika tidak mengkonsumsi daging<br>77. Lainnya. Sebutkan: ……….	[ ]	[FA35]
36.	Darimana sumber utama susu yang dikonsumsi keluarga Anda dalam 2 bulan terakhir?	1. Produksi sendiri<br>2. Pasar domestik<br>3. Pemberian<br>66. Tidak relevan jika tidak mengkonsumsi susu<br>77. Lainnya. Sebutkan: ……….	[ ]	[FA36]

Kecamatan		Desa		No. Responden		

FORM 24-hour FOOD RECALL

Nama Anak :

Data untuk Konsumsi Makan Hari/ Tanggal :

L. KONSUMSI MAKANAN ANAK

L1. Daftar Makanan yang Dikonsumsi Anak Dalam Satu Hari Kemarin

INDIVIDU RECALL 1 X 24 JAM (ANAK)

No.	Waktu Makan	Menu	Metode Memasak	Komposisi/ Bahan Makanan/ Brand	Berat Makanan	
					URT	Gram

Kecamatan		Desa		No. Responden		

***Jika masih ASI eksklusif, tulis "ASI" saja disertai jam-jam anaknya meminum ASI**

1	Status Menyusui	1. Ya	0. Tidak	[ ]	[BFstat]
2	Apakah konsumsi makanan dalam satu hari tersebut adalah makanan yang biasa di konsumsi sehari-hari?	1. Ya→ **lanjut ke no 3**	0. Tidak	[ ]	[UsualDiet]
2a	Jika tidak sama dengan kebiasaan makan selama ini, apa yang berbeda?<br>Jumlahnya, yaitu ....<br><br>66. Tidak relevan jika makanan merupakan makanan yang biasa dimakan				[UDfreq]
2b	Jika tidak sama dengan kebiasaan makan selama ini, apa yang berbeda?<br>Jenisnya, yaitu ....<br><br>66. Tidak relevan jika makanan merupakan makanan yang biasa dimakan				[UDtyp]
3	Apakah [nama anak] mengkonsumsi suplemen?	1. Ya (lanjut ke 3a)	0. Tidak	[ ]	[Supplmn]
3a	Jika iya, apa merknya?				[SupBrand]
3b	Berapa jumlah dan frekuensi yang dikonsumsi?				[SupFreq]

4 DDS (Dietary Diversity) 7 food groups
Catatan: Tidak perlu ditanyakan, data akan diambil dari recall 24 jam
<u>HANYA UNTUK ANAK DI ATAS 6 BULAN</u>

No	Kelompok Pangan	Contoh	Kode<br>1. Ya 0. Tidak<br>66. Tidak relevan jika masih ASI eksklusif dan <6 bulan	
1	Biji-bijian, akar-akaran, dan umbi-umbian	Jagung, beras, mie, biscuit, cookies, kentang putih, singkong	[ ]	[DDS1]
2	Sayur dan buah sumber Vitamin A	Waluh, wortel, ketela kuning, buah-buahan berwarna jingga terang	[ ]	[DDS2]
3	Buah dan sayuran lainnya	Buah dan sayur selain yang disebutkan di no 2	[ ]	[DDS3]
4	Daging dan ikan	Daging ayam, sapi, kambing, ikan, kerang, makanan laut, jeroan	[ ]	[DDS4]
5	Telur	Telur ayam, bebek, puyuh	[ ]	[DDS5]
6	Kacang-kacangan	Pangan dari kacang-kacangan	[ ]	[DDS6]
7	Susu dan produknya	Susu, keju, yoghurt dan olahan susu lainnya	[ ]	[DDS7]

5	Hasil Analisis Konsumsi Makanan (Nutrisurvey)		
5a	Energi		[Energi]
5b	Protein		[Protein]
5c	Karbohidrat		[CHO]
5d	Lemak		[Fat]
5e	Zinc		[Zinc]
5f	Iron		[Iron]
5g	Vitamin A		[VitA]
5h	Vitamin C		[Vit C]
5i	Asam Folat		[AsFolat]

Kecamatan	Desa	No. Responden
☐☐	☐☐	☐☐☐

P. Pengukuran Antropometri dan Hemoglobin Anak (0-11 bulan)		
Tanggal pengukuran	:	....
Nama anak	:	....
Nama ibu kandung	:	....
Jenis kelamin anak	:	Laki-laki / Perempuan* *pilih salah satu
Tanggal lahir anak	:	...... / …... / ......

1	**P1. Pengukuran Berat Badan**[CHweight]			
	a. Pengukuran 1	__ __, ___ kg	Maksimum beda pengukuran: **0.1 kg**	[BWChild1]
	b. Pengukuran 2	__ __ , ___ kg		[BWChild2]
	c. Nama Pengukur		[ ]	[MeasCHWeight]
2	**P2. Pengukuran Tinggi Badan**[CHlength]			
	Posisi	Telentang	Maksimum beda pengukuran:<br>Panjang Badan= **0.7 cm**	
	a. Pengukuran 1	___ ___ ___ , ____ cm		[HChild1]
	b. Pengukuran 2	___ ___ ___, ____cm		[HChild2]
	c. Nama Pengukur		[ ]	[MeasCHLength]
3	**P3. Pengukuran Hb**[CHHb]			
	a. Pembacaan1	___ ___ , ___ g/dL	*Cut off point non-Anemia* : $\geq$11 g/dL<br>**Maksimum beda pengukuran : 0.2 g/dL**	[HbChild1]
	b. Pembacaan 2	___ ___ , ___ g/dL		[HbChild2]
	c. Nama Pengukur		[ ]	[MeasCHHb]

OBSERVASI DAN CATATAN:

Kecamatan	Desa	No. Responden
☐☐	☐☐	☐☐☐

Q. Pengukuran Antropometri dan Hemoglobin Ibu Kandung	
Tanggal pengukuran	: ....
Nama ibu kandung	: ....
Tanggal lahir ibu	: ...... / …... / ......

1	**Q1. Pengukuran Berat Badan**[MOMWeight]			
	a. Pengukuran 1	__ __, ___ kg	Maksimum beda pengukuran: 0.1 kg	[BWMom1]
	b. Pengukuran 2	__ __ , ___ kg		[BWMom2]
	c. Nama Pengukur		[ ]	[MeasMOMWeight]
2	**Q2. Pengukuran Tinggi Badan**[MOMHeight]			
	a. Pengukuran 1	___ ___ ___ , ____ cm	Maksimum beda pengukuran:<br>Tinggi Badan = **0.7 cm**	[HMom1]
	b. Pengukuran 2	___ ___ ___, ____cm		[HMom2]
	c. Nama Pengukur		[ ]	[MeasMOMHeight]
3	**Q3. Pengukuran Hb**[MOMHb]			
	a. Pembacaan1	___ ___ , ___ g/dL	**Cut off point non-Anemia wanita tidak hamil : >12 g/dL**<br>**Cut off point non-Anemia wanita hamil : >11 g/dL**<br>**Maksimum beda pengukuran : 0.2 g/dL**	[HbMom1]
	b. Pembacaan 2	___ ___ , ___ g/dL		[HbMom2]
	c. Nama Pengukur		[ ]	[MeasMOMHb]

OBSERVASI DAN CATATAN :

Kecamatan | Desa | No. Responden

PANDUAN GAMBAR UNTUK PROBING KUESIONER

WC

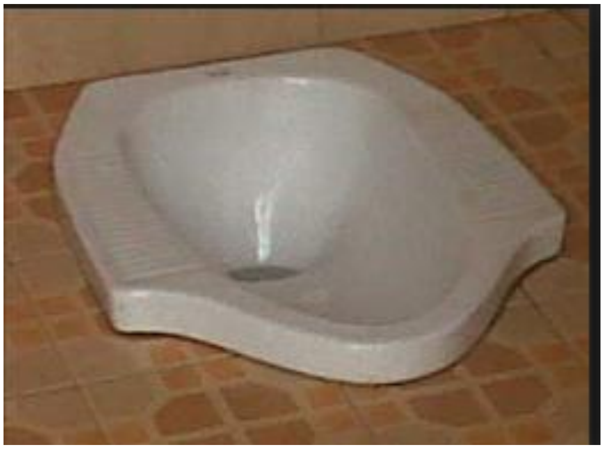

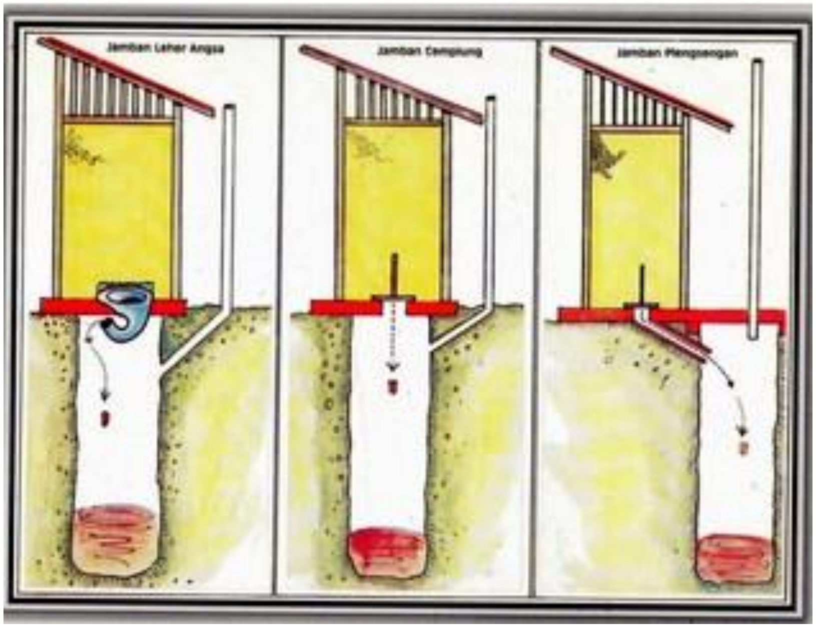

LEHER ANGSA | CEMPLUNG | PLENGSENGAN

KAPSUL VITAMIN A

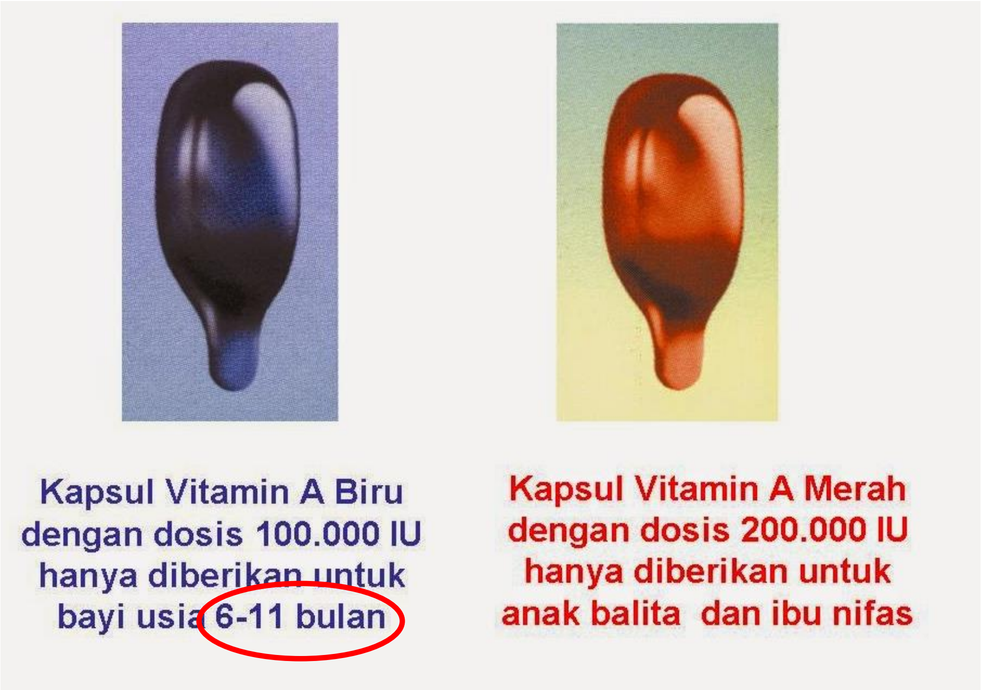

Supplement: S1 Questionnaire — (PDF) [file pone.0254662.s001.pdf]
