# Supplementary material for: Prenatal and postnatal determinants of stunting at age 0–11 months: A cross-sectional study in Indonesia
Source: PLoS One. 2021 Jul 14;16(7):e0254662. doi: 10.1371/journal.pone.0254662 (PMC8279365; doi:10.1371/journal.pone.0254662)
Supplement: Attachment — Submitted filename: PONE-D-20-10544_Determinants of stunting among infants aged 0-11 months.docx [file pone.0254662.s002.docx]

**Determinants of Stunting among Infants Aged 0-11 Months in a Borderline Area of Indonesia: Evidence from Sambas longitudinal study on Maternal and Child Nutrition, Indonesia**

**Title:** The title is too long and its length needs to be reduced.

- For example: “Prenatal and postnatal determinants of stunting at age 0-11 months: A cross-sectional study in Indonesia”

**Abstract:**

- Background:
  - Too long, as it includes information that should go into the methods section.
  - For example, following the sentence ………. adult life, authors could simply write: “we conducted a longitudinal study on maternal and child nutrition to assess/investigate factors associated with stunting among infants aged 0-11 months in Indonesia”
- Methods:
  - It is not clear how this was a longitudinal study.
  - Did the study enroll pregnant women in 2016? If yes, how many and what was the gestational age at enrollment?
  - Did the 2017 survey include the same mothers who were previously enrolled in the study in 2016 and infants born from those index pregnancies?
  - When was the anthropometric data collected?
- Results:
  - Authors should always report both the percentage and the corresponding number (n).
  - Authors should also report on the key characteristics of studied mothers and children before reporting on the factors associated with stunting.
  - “Furthermore, children who weighed <2.500 g at birth had the strongest
    association with stunting”. Authors should consider deleting this sentence from the results section and probably put it into the conclusion, as it looks like the interpretation of the already reported result.
- Conclusion:
  - This section should be improved. Authors are responsible for interpreting the key findings from this study and thereby suggest (what) interventions (by who?) to address the problems identified to be associated with stunting in infancy (0-11 months).

**Manuscript:**

- Methods:
  - Major comment: This study was not a longitudinal study, as the outcome of interest (stunting) was only measured at one time-point. This was a cross-sectional study with some retrospective data during pregnancy.
  - Authors’ calculations indicated that a sample n=492 (lines 129-135) was sufficient to conduct this study. But, was this the number of children or mothers? Was it necessary to calculate a sample size if this was a follow up survey that should include all subjects found?
  - In the sampling procedure (lines 137-142), authors state that 30 villages were randomly sampled, but out of how many? Why 30 villages? Why did you have to sample?
  - Instruments (lines 143-150), this should be part of the data collection procedure, and authors should avoid repetition.
  - Variables and operational definition:
    - Minimum dietary diversity and acceptable diet – as this study only included infants aged 0-11 months, how did you categorize children under 6 months of age?
    - Minimum meal frequency – what was the minimum number of meals? And how did you classify under 6 months infants?
    - Household food security: It’s good to provide the general definition of food security, but how did authors define “household food security” in this particular study?
    - How was good/poor knowledge of IYCF defined?
  - Data quality assurance – how was it possible to check for under- and/or over-reporting in dietary diversity data during data cleaning? Please explain!
  - Data collection period – Is it April 2^nd^ – May 17, 2017?
  - Statistical analysis:
    - Please use the past tense.
    - Is it true that the multivariable model included variables with a p>0.25 in bivariate analysis? Please double-check if it was not a typo!
    - Please clarify the unit of analysis for this study! Is it children/mothers?
    - Child age and sex are factors that have been consistently found to be associated with stunting and should be forced into the final logistic regression model regardless of their p-values.
- Results:
  - Lines 209-212. This paragraph should go under the “study settings” in the Methods.
  - Please avoid interpretation of results in the “Results section” (example, lines 233-234). All interpretations should go under the “Discussion section”.
  - It is surprising that the results’ section focused on participants’ characteristics rather than reporting on the key findings (factors associated with stunting) in line with the study objectives. Lines 281-284, please include the odds ratio to highlight the adjusted effect size of each factor on stunting.
  - The number of tables (13) is more than acceptable for a research article. Please consider reducing the number of tables:
    - Authors could use a cross-tabulation for:
      - Table 2: Child characteristics and stunting
      - Table 3: Maternal/Caregiver characteristics and stunting
      - Table 4: Household characteristics and stunting
      - Table 5: Multivariable logistic regression model results
  - Old Table 5 is not needed. A composite variable for “Good/poor knowledge of IYCF” should only be reported in the new suggested table (Table 3).
  - Results’ tables should only include data on participants who were found in the 2017 survey. Therefore, the sample size should be less or equal to 500 (n≤500).
- Discussion:
  - A classical discussion should focus on the key findings in terms of prenatal and postnatal factors associated with stunting in the multivariable logistic regression analysis. Authors should avoid discussing anything which is not supported by the findings of this study.
  - A stunting rate of 22.5% among children aged 6-11 months was very high, and worth discussing.
  - Study limitations:
    - Lines 583-584. What do you mean by the technical error?
    - Second, authors said in the methods that a sample size, n=492 was sufficient to conduct this study. Fortunately, 500 children were found for this study, so how does this become a limitation?
  - Conclusions:
    - Conclusions should be based on the findings of this study. Authors are responsible for suggesting solutions.
