# Supplementary material for: Prenatal and postnatal determinants of stunting at age 0–11 months: A cross-sectional study in Indonesia
Source: PLoS One. 2021 Jul 14;16(7):e0254662. doi: 10.1371/journal.pone.0254662 (PMC8279365; doi:10.1371/journal.pone.0254662)
Supplement: Attachment — Submitted filename: Response to Reviewers.docx [file pone.0254662.s004.docx]

Dear Reviewers,

Thank you for giving us the opportunity to improve and resubmit our manuscript “PONE-D-20-10544R1: Prenatal and postnatal determinants of stunting at age 0–11 months: A cross-sectional study in Indonesia”. Please find enclosed the revised manuscript for further consideration. The manuscript has been revised according to the comments raised by the reviewer. We would like to thank the reviewer for the constructive and competent criticism, and we hope that our manuscript will be acceptable for publication in PLOS ONE.

A. **Additional Editor Comments (if provided):**
B. Heading: Consider replacing “stunting at 1 age 0–11 months” with “stunting among children aged 0–11 months”

**RESPONSE:**

Thank you for your suggestion. We have replaced “stunting at age 0-11 months” with “stunting among children aged 0-11 months”.
2. The statistical analysis section still needs to be improved.

**RESPONSE:**

Thank you for providing these insights. We have amended the statistical analysis section to be more comprehensive and clearer.
3. The first paragraph under Statistical analysis is not well written and there is plenty of room for improvement. Please pay attention to the logical steps in data analysis. For example, Mantel–Haenszel analysis is usually done before multivariable logistic regression.

**RESPONSE:**

Thank you for providing these insights. As we mentioned before, we have amended the statistical analysis section including the logical steps in data analysis. We agree that Mantel-Haenszel analysis should be done before multivariable logistic regression.
4. Line 180: What is the difference between “participant’s characteristics” and “variables included in this study”?

**RESPONSE:**

Thank you for your question. We have put the explanation for the difference between “participant’s characteristics” and “variables included in this study” as follow:

a. “Participant’s characteristics” were variables which refers to socio-demographic variables such as: gender, educational background of the mother’s, family income and etc.
b. “Variables includes in this study” refers to prenatal and post-natal variables including: Prenatal variables: maternal short stature, maternal CED risk, knowledge during pregnancy etc.

Post-natal variables: morbidity, birth condition and utilization of health services.

Please look at Table 1. Variables and Operational Definition, we have included participant’s characteristics in the table.

5. This sentence “To analyze normal distribution of data, the Kolmogorov–Smirnov Z test was used with a cut off point p < 0.05” does not make sense. Which variables were assessed? What is the meaning of “analyze normal distribution”?

**RESPONSE:**

Thank you for raised this concern. We agree that the sentence for normal distribution should be revised. Following your suggestion, we deleted this sentence since they weren’t non parametric test that we used in this study.
6. You mention that “Mantel–Haenszel analysis was performed to control for confounding.” It is not clear which confounding variables were adjusted for. Moreover, M-H methods can only adjust for one variable. Where are results of this analysis? I think you used multivariable logistic regression to adjust for confounding. Use of M-H methods is insufficient to adjust for confounding by many factors.

**RESPONSE:**

Thank you for providing these insights. We agree with you that M-H only adjust for one variable and multivariable logistic regression is suitable and correct analysis to adjust for confounding by many factors.

7. Table 2 heading: replace “conditions” with “characteristics”

**RESPONSE:**

We agree with you. We have replaced “conditions” with “characteristics”.

8. As mentioned in my previous comments, collapse some categories with few cases (for example ethnicity).

**RESPONSE:**

Thank you for your input. We have collapsed some categories.
9. Table 2: Delete the subheading “Economic Condition”.

**RESPONSE:**

We deleted the sub heading economic condition. Thank you

10. For household income and expenditure, it is unclear whether you have presented frequencies and percentages or medians. For example, this “≥2,000,000* (median)” is not clear.

**RESPONSE:**

Thank you for insights. We have amended household income and expenditure variables to be clear. Those variables were presented as frequencies and percentage based on median cut off.
11. Present odds ratios with 95% CIs to 2 decimal points.

**RESPONSE:**

Thank you for your suggestion. We have amended odds ratios with 95% Cis to 2 decimal points.
12. Since you have indicated the exact p values, there is no need to include the footnote “*=significant p<0.05”. This footnote is redundant.

**RESPONSE:**

Yes, we agree with you. We have deleted the footnote accordingly.
13. In the tables, P values of 0.000 should be written as <0.001. This has already been pointed out by one of the reviewers.

Thank you for your input. We have revised the P values of 0.000 and written as <0.001

**RESPONSE:**
14. In multivariable analysis, indicate what has been adjusted for what. You can do this under statistical analysis or in the footnotes of the tables.

**RESPONSE:**

Thank you for your input. We have put the explanation for adjusted variables in the footnote.

15. Note that low birth weight is an obvious mediator between maternal/household characteristics and stunting, therefore it should not be adjusted for when assessing the effect of these factors on stunting. For example, anemia during pregnancy → Low birth weight →Stunting. In this case, stunting is on the causal pathway and should not be adjusted for.

**RESPONSE:**

Thank you for your input. We have amended the analysis where LBW should not be adjusted when assessing the effect of these factors on stunting.
16. In the stunting column in the tables, you can write Stunted with the options of Yes and No. Alternatively, simply indicate the % stunted. This will make the table neater.

**RESPONSE:**

Thank you for your suggestion. We have revised the stunting column in tables.

17. In the table headings, please write “Association between A and B. For example, Table 3. “Association between characteristics of children and stunting”

**RESPONSE:**

Thank you for your input. We have amended the table headings.

18. In the discussion, delete the subheading “Key Findings” because the text under this subheading goes beyond that.

**RESPONSE:**

Thank you for your input. We deleted the subheading “Key Findings”.

19. Overall, the manuscript should be proofread to ensure it meets the journal’s English language requirements.

**RESPONSE:**

Thank you for your suggestion. As we stated in the first revision, we already proofread our manuscript to Enago Proofreading where the certificate was also attached in the journal platform.

**B.Reviewers' comments to the Author**

Reviewer #1: The authors have addressed most of my earlier concerns well. I have a few other minor comments for their consideration.
**RESPONSE:**

Thank you very much for your valuable inputs and correction. We really appreciate the time and effort you and each of the reviewers have dedicated to providing insightful feedback on ways to strengthen our paper.

1. Abstract, line30; <2.500g, should be checked and written correctly (<2500g or <2.500kg).

**RESPONSE:**

Thank you for your input. The correct category for LBW is <2500 gram or 2.5 kilogram.

2. Methods section, statistical analysis: The bivariate analysis are described well. Authors should add some details about the multivariable modelling performed. Did authors perform only Mantel-Haenszel (MH) analysis for controlling confounding? Clarify which factors are adjusted in the reported adjusted odds ratios. I think the MH analysis is less suitable to handle beyond 3 variables in a single model. If logistic regression was used, please describe this in the methods.

**RESPONSE:**

Thank you for your input. We have amended the MH analysis in our paper. We didn’t include MH analysis since we used multivariable logistic regression for adjusted more than 3 variables.

3. Page 27, line 304: references 18 and 19 need a comma separation.

Thank you for your input. We have revised the references 18 and 19.

Reviewer #3: Many congratulations to authors - so much effort has been made to improve this paper!! However, there are still remaining minor comments which I hope addressing them can make the paper look even better.

**RESPONSE:**

Thank you very much for your valuable inputs and correction. We really appreciate the time and effort you and each of the reviewers have dedicated to providing insightful feedback on ways to strengthen our paper.

1. Please revise the abstract's conclusion, where you say that ONLY postnatal factors were significantly associated with stunting and none of the prenatal factors was statistically associated with stunting. In your logistic regression model, factors associated with stunting included low birth weight, a result from the intra-uterine growth restriction and it should be considered as a pre-natal factor.

**RESPONSE:**

Thank you for your suggestion. We have revised the abstract’s conclusion that low birth weight, a result from the intra-uterine growth restriction and it should be considered as a pre-natal factor.

2. The manuscript overall conclusion is vague - and this conclusion is not supported by the results. Authors say that several prenatal and postnatal factors were significantly associated with stunting, while only three factors (LBW, diarrhea and complete immunization) were important?

**RESPONSE:**

Thank you for your input. We have amended the manuscript specially to give more clear result from our study.
