# Supplementary material for: Prenatal and postnatal determinants of stunting at age 0–11 months: A cross-sectional study in Indonesia
Source: PLoS One. 2021 Jul 14;16(7):e0254662. doi: 10.1371/journal.pone.0254662 (PMC8279365; doi:10.1371/journal.pone.0254662)
Supplement: Attachment — Submitted filename: Response to Reviewers.docx [file pone.0254662.s005.docx]

Dear Reviewers,

Thank you for giving us the opportunity to improve and resubmit our manuscript “PONE-D-20-10544R1: Prenatal and postnatal determinants of stunting at age 0–11 months: A cross-sectional study in Indonesia”. Please find enclosed the revised manuscript for further consideration. The manuscript has been revised according to the comments raised by the reviewer. We would like to thank the reviewer for the constructive and competent criticism, and we hope that our manuscript will be acceptable for publication in PLOS ONE.

**Additional Editor Comments**

Based on editor and reviewers’ suggestions, I would like to inform you some minor revision as you mentioned in the previous email:

1. Although you have responded that “We have put the explanation for adjusted variables in the footnote”, I could not locate this.

Response: We have put the explanation for adjusted variables in the footnote. Thank you for your concern.

2. You have also responded that “We have amended the analysis where LBW should not be adjusted when assessing the effect of these factors on stunting.” Does this mean you re-analyzed the data after excluding birthweight from the model? If this is the case, then it is surprising that the effect estimates, 95% CIS and p-values have not changed (at least this is not marked on the version with tracked changes).

Response: We have amended the analysis where LBW should not be adjusted. Thank you for reminded us.

3. In line with the above, please ensure all responses to the previous comments are reflected in the revised manuscript to avoid further delays.

Response: Thank you for your suggestion. We have ensured all responses were reflected in the revised manuscript.

4. The manuscript still has some grammatical errors that need to be revised to meet the journal’s English language requirement. I cannot identify all the errors, so please review the manuscript carefully. Below are examples.

The statement below under “Statistical Analysis” describes what will be done. Please change the tense to reflect what was done (note that “Categorical data will be applied...” does not make sense).

“Categorical data will be applied in a frequency table to display its frequency and percentage. Numeric data will also be reported as mean scores and standard deviations if the collected data is normally distributed. Meanwhile, if the collected data is not normally distributed, it will be presented as median and minimal-maximum value”

Response: Thank you for your suggestion. We have revised your suggestions in the revised manuscript.

5. The heading of Table 2 is missing the word “characteristics” after “socio-demographic”

Response: Thank you for your suggestion. We have revised in the revised manuscript.

6. For this statement “Children with low birth weight had higher odds ratio (ORs) of being stunted” it is correct to say “Children with low birth weight had higher odds of being stunted…”

Response: Thank you for your suggestion. We have revised in the revised manuscript.

We have incorporated changes that reflect the detailed suggestions you have graciously provided. We also hope that our edits and the responses we provide below satisfactorily address all the issues and concerns you and the reviewers have noted.
