# Supplementary material for: Prenatal and postnatal determinants of stunting at age 0–11 months: A cross-sectional study in Indonesia
Source: PLoS One. 2021 Jul 14;16(7):e0254662. doi: 10.1371/journal.pone.0254662 (PMC8279365; doi:10.1371/journal.pone.0254662)
Supplement: Attachment — Submitted filename: Response to Reviewers3.docx [file pone.0254662.s006.docx]

Dear Reviewers,

Thank you for giving us the opportunity to improve and resubmit our manuscript “PONE-D-20-10544R1: Prenatal and postnatal determinants of stunting at age 0–11 months: A cross-sectional study in Indonesia”. Please find enclosed the revised manuscript for further consideration. The manuscript has been revised according to the comments raised by the reviewer. We would like to thank the reviewer for the constructive and competent criticism, and we hope that our manuscript will be acceptable for publication in PLOS ONE.

**Additional Editor Comments (April 2021)**

Based on editor and reviewers’ suggestions, I would like to inform you some minor revision as you mentioned in the previous email:

1. Although you have responded that “We have put the explanation for adjusted variables in the footnote”, I could not locate this.

**Response:** We have put the explanation for adjusted variables in the footnote. Thank you for your concern.

2. You have also responded that “We have amended the analysis where LBW should not be adjusted when assessing the effect of these factors on stunting.” Does this mean you re-analyzed the data after excluding birthweight from the model? If this is the case, then it is surprising that the effect estimates, 95% CIS and p-values have not changed (at least this is not marked on the version with tracked changes).

**Response:** We have amended the analysis where multivariable analysis divided into 2 models. Model with LBW and without LBW. Thank you for reminded us.

3. In line with the above, please ensure all responses to the previous comments are reflected in the revised manuscript to avoid further delays.

Response: Thank you for your suggestion. We have ensured all responses were reflected in the revised manuscript.

4. The manuscript still has some grammatical errors that need to be revised to meet the journal’s English language requirement. I cannot identify all the errors, so please review the manuscript carefully. Below are examples.

The statement below under “Statistical Analysis” describes what will be done. Please change the tense to reflect what was done (note that “Categorical data will be applied...” does not make sense).

“Categorical data will be applied in a frequency table to display its frequency and percentage. Numeric data will also be reported as mean scores and standard deviations if the collected data is normally distributed. Meanwhile, if the collected data is not normally distributed, it will be presented as median and minimal-maximum value”

Response: Thank you for your suggestion. We have revised your suggestions in the revised manuscript.

5. The heading of Table 2 is missing the word “characteristics” after “socio-demographic”

Response: Thank you for your suggestion. We have revised in the revised manuscript.

6. For this statement “Children with low birth weight had higher odds ratio (ORs) of being stunted” it is correct to say “Children with low birth weight had higher odds of being stunted…”

Response: Thank you for your suggestion. We have revised in the revised manuscript.

**Additional Editor Comments (May, 2021)**

1. My previous comments were not adequately addressed. It is insufficient to say that you have revised the manuscript without stating how/where you revised or which change you made.

**Response**: Thank you for your concern. Apologize for the mistake. I have put the wrong file in the manuscript portal. In the latest revision, I already put the manuscript with track changes.

2. Please include a clean copy and a copy with tracked changes. It is difficult to see where you made changes.

**Response:** Thank you for your concern. Apologize for the mistake. I have put the wrong file in the manuscript portal. In the latest revision, I already put the manuscript with track changes.

3. Although you have mentioned in the footnotes that you adjusted for child, maternal and household characteristics, this is too generic and will not inform the readers which specific variables you adjusted for. Although birth weight should not be adjusted for because it is an intermediate variable, it can be assessed in a separate model as an independent predictor of stunting.

**Response:** Thank you for your suggestion. We already put the specific variables for adjusted variables that we used in the model.

4. The manuscript still has grammatical/spelling errors. It seems like new errors were introduced during English editing. For example, May was changed to mei (lines 99, 185), which is incorrect. Please review the entire manuscript for grammatical errors/typos.

**Response:** Thank you for your input. We revised the error/typos in our manuscript.

5. Change the sub-heading “Population and Subject” to “Study Population and Subjects”. In the previous version, this was written in continuous prose, which I think was a better format than the revised one.

**Response:** Thank you for your input. We already revised the error/typos in our manuscript.

6. Some statements in the methods are still in the future tense. For example this statement under the Statistical analysis section “Meanwhile, if the collected data were not normally distributed, it will be presented as median and minimal-maximum value.” Also, note that the median is usually presented with an interquartile range.

**Response:** Thank you for your suggestion. We already put interquartile range in our manuscripts (statistical analysis section and result section).

7. Check the tense in these sentences too: “Using pairwise deletion, the number of cases will vary. It will be the number of cases where both variables in the correlation are present”. The second sentence is not clear. What will be?

**Response:** Thank you for your concern. After we elaborated the method, we revised the method. Pairwise deletion was changed to listwise deletion. We run the analysis who had complete cases.

8. This statement is still not clear “Categorical data were applied in a frequency table to display its frequency and percentage.” You could say you summarized categorical data using frequencies and percentages, or something like that.

**Response:** we have revised the statement. Thank you for your input.

9. Use “multivariable analysis” instead of “multivariate analysis”

**Response:** Thank you for your suggestion. We changed it from multivariate to multivariable analysis.

10. “A significant association was determined at a p value <0.05, at α = 5%.” What is the difference between the two values? α = 5% seems like a repetition. Consider whether you need this statement because you have stated on line 197/198 that “For all tests, a P-value < 0.05 was considered statistically significant.”

**Response:** Thank you for your suggestion. We have revised the statement.

We have incorporated changes that reflect the detailed suggestions you have graciously provided. We also hope that our edits and the responses we provide below satisfactorily address all the issues and concerns you and the reviewers have noted.
